# Intraband transitions at a CsPbBr_3_/GaAs heterointerface in a two-step photon upconversion solar cell

**DOI:** 10.1038/s41598-024-78257-x

**Published:** 2024-11-06

**Authors:** Hambalee Mahamu, Shigeo Asahi, Takashi Kita

**Affiliations:** Department of Electrical and Electronic Engineering, Graduate School of Engineering, Kobe University, 1–1 Rokkodai, Nada, Kobe, 657–8501 Japan

**Keywords:** Solar cells, Devices for energy harvesting, Nonlinear optics, Electronic properties and materials, Surfaces, interfaces and thin films

## Abstract

Two-step photon upconversion solar cells (TPU-SCs) based on III–V semiconductors can achieve enhanced sub-bandgap photon absorption because of intraband transitions at the heterointerface. From a technological aspect, the question arose whether similar intraband transitions can be realized by using perovskite/III–V semiconductor heterointerfaces. In this article, we demonstrate a TPU-SC based on a CsPbBr_3_/GaAs heterointerface. Such a solar cell can ideally achieve an energy conversion efficiency of 48.5% under 1-sun illumination. This is 2.1% higher than the theoretical efficiency of an Al_0.3_Ga_0.7_As/GaAs-based TPU-SC. Experimental results of the CsPbBr_3_/GaAs-based TPU-SC show that both the short-circuit current *J*_SC_ and the open-circuit voltage *V*_OC_ increase with additional illumination of sub-bandgap photons. We analyze the excitation power dependence of *J*_SC_ for different excitation conditions to discuss the mechanisms behind the enhancement. In addition, the observed voltage-boost clarifies that the *J*_SC_ enhancement is caused by an adiabatic optical process at the CsPbBr_3_/GaAs heterointerface, where sub-bandgap photons efficiently pump the electrons accumulated at the heterointerface to the conduction band of CsPbBr_3_. Besides the exceptional optoelectronic properties of CsPbBr_3_ and GaAs, the availability of a CsPbBr_3_/GaAs heterointerface for two-step photon upconversion paves the way for the development of high-efficiency perovskite/III–V semiconductor-based single-junction solar cells.

**Supplementary Information:**

The online version contains supplementary material available at 10.1038/s41598-024-78257-x.

## Introduction

Photovoltaics is the field of science concerned with the conversion of photons to electrical energy. To characterize the physics of a considered photovoltaic device, usually, several parameters including the energy conversion efficiency (the ratio of the output electrical power to the power provided by the incident light) are used. The energy conversion efficiency of a solar cell (SC) can be estimated using thermodynamic approaches by considering the energy generation in the SC through absorption and the energy losses during the energy conversion process. The Shockley–Queisser (SQ) limit, a widely recognized mathematical model for SC simulation, predicts that the maximum conversion efficiency of a single junction SC is ~ 31% under 1-sun illumination^1–4^. To overcome the SQ limit, a SC design is needed that manipulates a part of the energy losses and suppresses them. For example, various SC designs have been proposed to reduce the transmission loss^5–7^, which plays an important role in SCs and is usually due to the small absorption coefficient of semiconductors for sub-bandgap photons.

In 1997, the concept of the intermediate-band solar cell (IBSC) was proposed to harvest energy in the region below the bandgap of the absorber layer of a single-junction SC^8^. An IBSC is equivalent to a parallel circuit: one branch contains one diode corresponding to band-to-band transitions (electronic transitions from the valence band (VB) to the conduction band (CB)), and the other branch contains two diodes connected in series corresponding to the sequential absorption of sub-bandgap photons via the intermediate band (IB) that exists between the CB and the VB. As a result, an IBSC is not affected by current and voltage mismatches, which are problematic in the case of multi-junction solar cells^1^. However, for IBSCs, a low density of states (DOS) at the IB levels is problematic, because this leads to weak carrier generation via the IB. Moreover, regarding the aspect of fabrication complexity, an IB needs a periodic long-range-ordered structure in order to create an electronic band. This requires complex fabrication processes, especially when the band is realized using quantum structures, e.g. stacked quantum dots (QDs). Such a trade-off relation between fabrication complexity and efficiency hinders the commercialization of IBSCs. On the other hand, we demonstrated a so-called two-step photon upconversion solar cell (TPU-SC) based on Al_0.3_Ga_0.7_As and GaAs including a single layer of InAs QDs at the heterointerface (the QDs were prepared by using the Stranski–Krastanov growth mode)^9–12^. The fabrication of such a TPU-SC is relatively simple compared to IBSCs.

A TPU-SC consists of a wide bandgap semiconductor (WGS) and a narrow bandgap semiconductor (NGS) to form a heterointerface, where intraband transitions can be induced by sub-bandgap photons. For the WGS and the NGS, we previously used Al_0.3_Ga_0.7_As and GaAs, respectively. The presence of QDs at the heterointerface can enhance the intraband transitions at the heterointerface, because the three-dimensional confinement of the QD relaxes the optical selection rule and allows in-plane electronic transitions^9,13–15^. Consequently, in addition to the band-to-band (interband) excitation of the WGS and the NGS, intraband excitation can be achieved, which results in a photocurrent enhancement: The process responsible for the photocurrent enhancement is called two-step photon upconversion (TPU), because each additional electron is first excited to the NGS CB by an interband-transition step and then experiences an intraband-transition step to reach the WGS CB. Regarding the photovoltage, optically induced intraband transitions lead to an increase of the quasi-Fermi level of the electrons due to an increase in the electron density of the WGS CB. Thus, the TPU-SC design is able to improve the photocurrent and the photovoltage, and therefore, the SC efficiency. The maximum theoretical efficiencies of a TPU-SC with a CB discontinuity at the heterointerface that is 60% larger than the VB discontinuity are 46.4% and 63.4% under 1-sun and 10,000-sun illumination respectively^1,16^. Therefore, the TPU-SC concept is one of the SC concepts that may help to realize high-efficiency single-junction SCs.

Although design concepts for SCs with high theoretical conversion efficiencies are very important, the actually used semiconductors are important as well. For example, lead halide perovskite semiconductors have been widely studied due to their excellent optoelectronic properties and advantages in terms of fabrication, since they can be prepared relatively easily using low-cost fabrication techniques^17–20^. Cesium lead halide perovskites (CsPbX_3_) show outstanding chemical stability with extraordinary optoelectronic properties. There are three main types of CsPbX_3_ perovskites: CsPbCl_3_, CsPbBr_3_, and CsPbI_3_ with energy bandgaps (*E*_g_) of 3.00 eV, 2.33 eV, and 1.73 eV respectively. Among those, CsPbBr_3_ demonstrates higher stability than CsPbI_3_ with a suitable *E*_g_ compared CsPbCl_3_ allowing CsPbBr_3_ to be a good candidate for device applications^20–23^. It has been reported that single-crystal CsPbBr_3_ films exhibit a high electron mobility of 1000 cm^2^ (Vs)^–1^ with a lifetime of 2.5 ms^20,24,25^. The carrier diffusion length, which influences the carrier collection efficiency in a SC, is approximately 1 μm and 12 μm for electrons and holes, respectively^26^, and the open-circuit voltage of CsPbBr_3_-based SCs can exceed 1.6 V^22,27^. In addition, CsPbBr_3_ has a high melting temperature of 570 °C, which means a relatively low degradation rate at room temperature. CsPbBr_3_ can form several perovskite crystal phases including the cubic *α*-phase, the tetragonal *β*-phase, and the orthorhombic *γ*-phase, depending on the environmental temperature^28–30^. Although CsPbBr_3_ exhibits three different crystal phases, their differences in terms of optoelectronic properties are not significant, and thus this material can be used in SCs over a wide range of operating temperatures^20,28^. The extraordinary good optoelectronic properties are promising for the realization of high-efficiency SCs. New technological insights might be gained by fabricating a TPU-SC based on CsPbBr_3_ and GaAs to study the potential of the CsPbBr_3_/GaAs heterointerface for TPU-SCs and intraband transitions in general.

In this work, we fabricated a TPU-SC with CsPbBr_3_ and GaAs as the WGS and the NGS, respectively, and confirmed enhancements in the photocurrent and the photovoltage in the case of additional illumination with sub-bandgap photons. The wide bandgap of CsPbBr_3_ (2.33 eV) is suitable for application in a TPU-SC together with GaAs (1.42 eV) due to the achieved ratio of the band discontinuities in the CB and VB^1,16^. We used a QD-free structure to study the effect of the interface states on the TPU process. Furthermore, we simplified the fabrication technique; the growth of CsPbBr_3_ was performed under ambient conditions. Although our CsPbBr_3_/GaAs-based TPU-SC contains no QDs, we believe that the interface states behave like quantized electronic states, which allow intraband transitions if the SC is irradiated with sub-bandgap photons in addition to the light used for interband excitation of the absorber layers in this device. The excitation power-dependence of the short-circuit current and that of the open-circuit voltage revealed the carrier dynamics at the CsPbBr_3_/GaAs-heterointerface as well as the quasi-Fermi-level splitting as a result of a TPU process. The effect of the temperature on the photocurrent and the photovoltage was investigated to distinguish the TPU process from thermal activation.

## Results and discussion

### Device structure of the TPU-SC

**Fig. 1:**
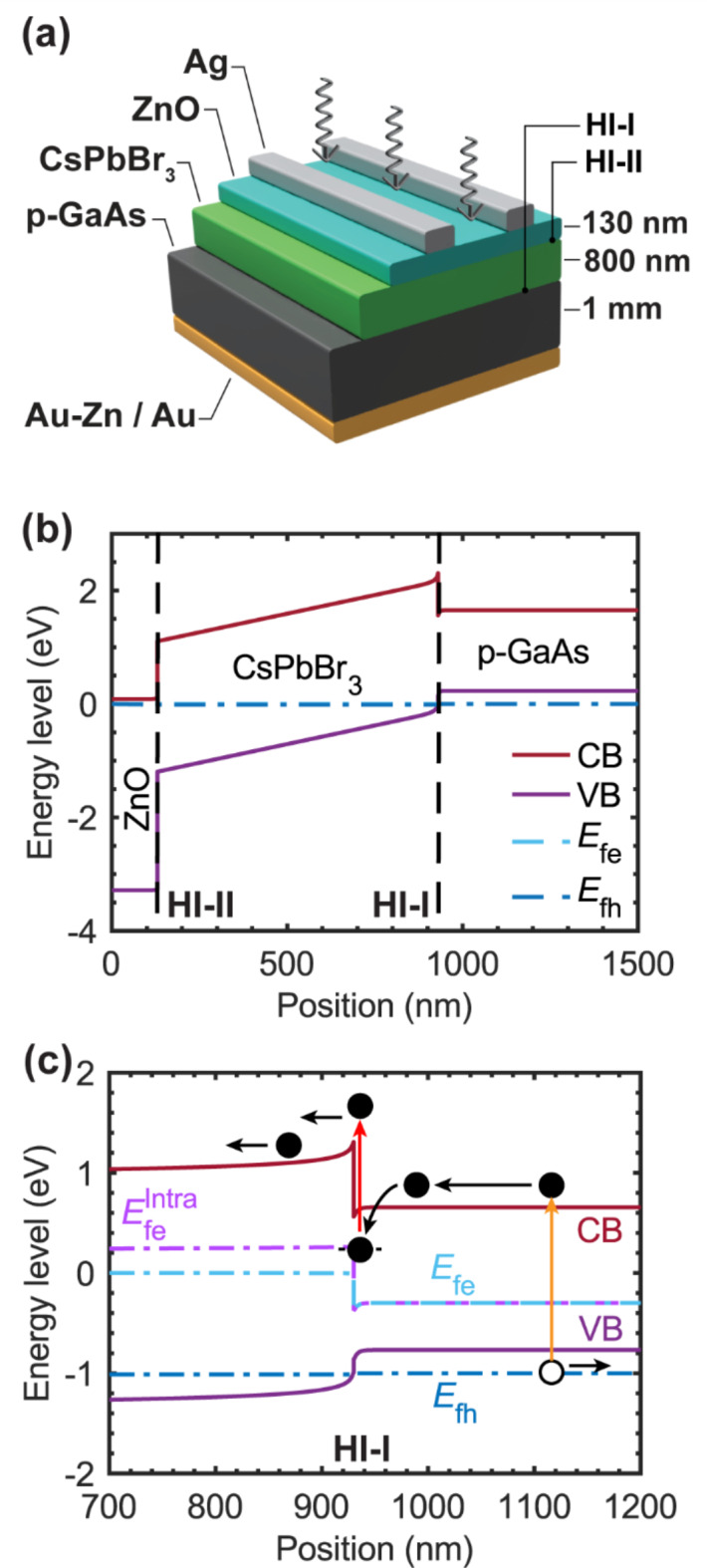
Schematic illustration of the CsPbBr_**3**_/GaAs-based TPU-SC and its band diagram. (**a**) The structure of the TPU-SC fabricated in this work. The *p*-GaAs substrate serves as the *p*-layer (the hole transport layer), and ZnO serves as the *n*-layer (the electron transport layer) in this device. (**b**) The energy band diagram of this device at thermal equilibrium conditions obtained using the COMSOL Multiphysics software. CB, VB, *E*_fe_ and *E*_fh_ in the legend stand for the conduction band, the valence band, and the quasi-Fermi levels of electrons and holes respectively. The CsPbBr_3_/GaAs and ZnO/CsPbBr_3_ heterointerfaces are indicated by the dashed lines with the labels HI-I and HI-II, respectively. (**c**) The TPU mechanisms occurring at HI-I depicted under the operating conditioned band diagram. The yellow arrow and red arrow correspond to the interband and intraband photoexcitations respectively.

The fabricated TPU-SC consists of an *n*–*i*–*p* CsPbBr_3_/GaAs heterojunction. The device structure is shown in Fig. 1a. For the WGS, we grew a CsPbBr_3_ layer (shown in green), and for the NGS, we used a 1-inch *p*-doped GaAs substrate (001) (shown in black). CsPbBr_3_ is an intrinsic semiconductor and *p*-GaAs is a hole transport material respectively. Although CsPbBr_3_ can possess *n*-type behaviors depending on self-doping of (PbBr_3_)^–34^, we regard CsPbBr_3_, in this work, as an intrinsic semiconductor as regarded by literature^31–33^. According to Ref.^34^, *n*-type behaviors can be observed when PbBr_2_ concentration is more than twice of CsBr due to self-doping effects. However, such self-doping effects are under debate. Ref.^18^ reported a fabrication recipe that used nearly similar precursor concentrations to our recipe. The researchers also regard CsPbBr_3_ as an intrinsic semiconductor absorber. Zinc oxide (ZnO) was used as the electron transport layer (ETL). We denote the CsPbBr_3_/GaAs heterointerface as HI-I, and the ZnO/CsPbBr_3_ heterointerface as HI-II. Therefore, our TPU-SC device structure is *n*–*i*–*p* corresponding to ZnO/CsPbBr_3_/*p*-GaAs. The band diagram of the ZnO/CsPbBr_3_/*p*-GaAs structure was simulated using the COMSOL Multiphysics software, and the result is shown in Fig. 1b. A TPU process is expected to occur at HI-I. The TPU mechanisms are depicted in Fig. 1c. The dark solid circle and white empty circle represent an electron and a hole respectively. Both are generated by photoexcitation in GaAs represented by the yellow arrow. The presence of the photogenerated hole and electron in the VB and the CB causes quasi-Fermi-level splitting within the GaAs layer. The quasi-Fermi levels of electrons (*E*_fe_) and holes (*E*_fh_) are indicated by light blue and dark blue dashed lines respectively. The electron drifts toward the HI-I while the hole drifts in the opposite direction as a result of the built-in electric field. The existence of discrete interface states at HI-I (the black dashed line where a solid circle occupies) can cause electron accumulation. Therefore, with intraband excitation (the red arrow in Fig. 1c), the accumulated electron can transit to the CB of CsPbBr_3_. This process causes a change in the *E*_fe_ within CsPbBr_3_, indicated by the light purple dashed line (${{\text{E}}}_{\text{fe}}^{\text{Intra}}$) in Fig. 1c, which results in photocurrent and photovoltage enhancement. The details of the simulation and semiconductor parameters, used in COMSOL Multiphysics, are described in Supplementary Information (Section S1). It is noted that some semiconductor parameters are not actual values. Results of the TPU process occurring at HI-I are discussed in the following sections.

### The ideal energy conversion efficiency

**Fig. 2:**
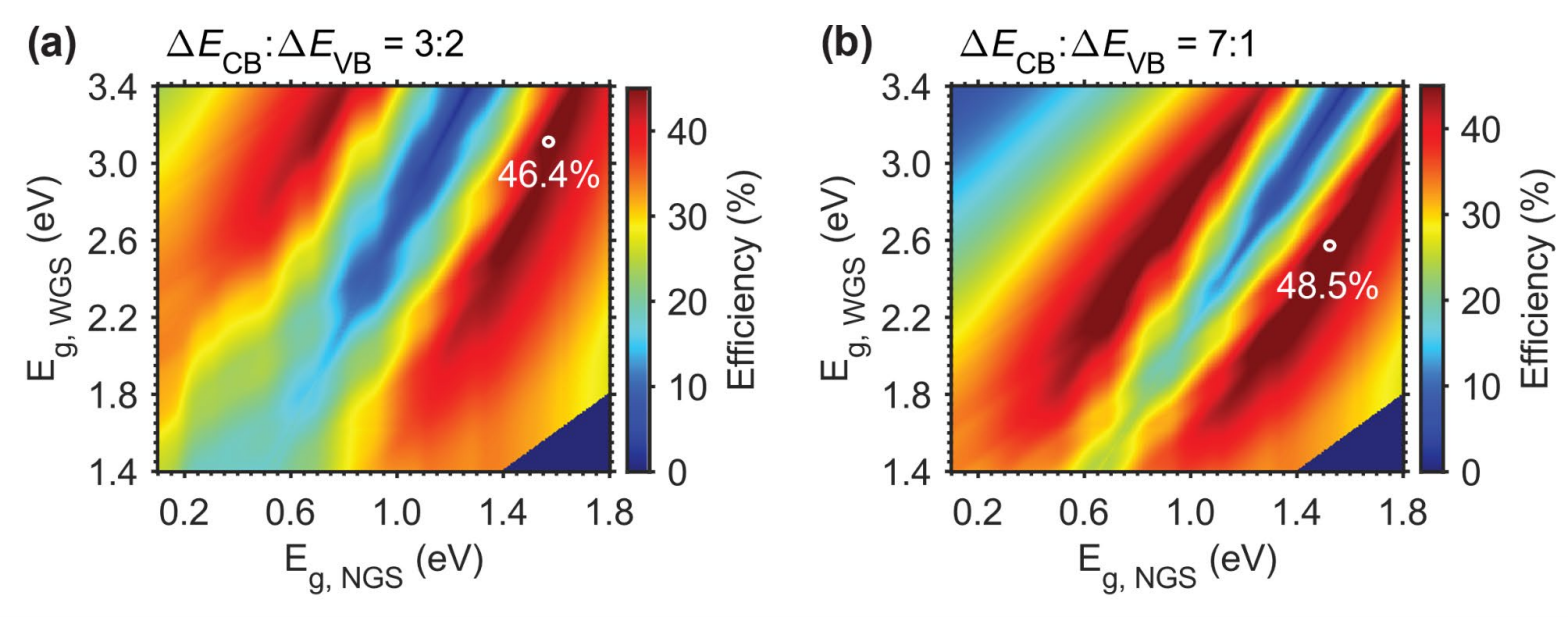
Two-dimensional maps of the ideal conversion efficiency. The maps show the ideal conversion efficiency under 1-sun illumination with the AM 1.5G spectrum for different ratios of the CB and VB discontinuities. (**a**) Results for Δ*E*_CB_:Δ*E*_VB_ = 3:2. (**b**) Results for Δ*E*_CB_:Δ*E*_VB_ = 7:1.

Figure 2 shows two maps of the theoretical conversion efficiency as functions of the NGS and WGS bandgaps for two different band-offset configurations. Here, the band-offset configuration is expressed by the ratio of the CB and VB discontinuities (Δ*E*_CB_:Δ*E*_VB_). For the prediction of the efficiency limit under 1-sun illumination, we used the detailed balance approach^1,9,16^ and the AM 1.5G spectrum. Figure 2a shows the data for Δ*E*_CB_:Δ*E*_VB_ = 3:2. This value of Δ*E*_CB_:Δ*E*_VB_ is nearly equal to that of an Al_0.3_Ga_0.7_As/GaAs heterojunction^16^. The highest conversion efficiency is 46.4%, which is achieved by using NGS and WGS bandgap energies of *E*_g, NGS_ ≈ 1.60 eV and *E*_g, WGS_ ≈ 3.10 eV, respectively. Therefore, GaAs (*E*_g_ = 1.42 eV) is a reasonable good choice for the NGS. However, the optimal WGS bandgap is very large, and thus Al_*x*_Ga_1-*x*_As is not the best choice for the WGS of such a high-efficiency TPU-SC (the bandgap energy of Al_*x*_Ga_1-*x*_As is only about 2.20 eV even for *x* = 0.9). Note that Al_0.3_Ga_0.7_As (*E*_g_ = 1.79 eV) was used in the previously reported TPU-SCs. Furthermore, although a larger Al molar fraction *x* provides a larger bandgap, Al_*x*_Ga_1-*x*_As switches from a direct bandgap semiconductor to an indirect bandgap semiconductor when *x* ≥ 0.45^35^.

The conversion efficiency map for Δ*E*_CB_:Δ*E*_VB_ = 7:1 is shown in Fig. 2b. Since the electron affinity (*χ*) and the *E*_g_ values of CsPbBr_3_ and GaAs are known, we can estimate the barrier heights for both the CB and the VB: The *χ* values of CsPbBr_3_ and GaAs are 3.30 eV and 4.07 eV, respectively^36,37^. Therefore, the ratio of Δ*E*_CB_ to Δ*E*_VB_ becomes 7:1 (Δ*E*_CB_ = 0.77 eV and Δ*E*_VB_ = 0.11 eV). Figure 2b indicates that the maximum conversion efficiency is 48.5% for *E*_g, NGS_ ≈ 1.50 eV and *E*_g, WGS_ ≈ 2.60 eV with a fill factor of 0.889. This efficiency maximum is 2.1% higher than that for Δ*E*_CB_:Δ*E*_VB_ = 3:2. Furthermore, we find that CsPbBr_3_ is suitable for the WGS of such a TPU-SC, because CsPbBr_3_ perovskite has an *E*_g_ of 2.33 eV. In comparison to the Al_0.3_Ga_0.7_As/GaAs-based TPU-SC, the CsPbBr_3_/GaAs-based TPU-SCs provide a higher maximum conversion efficiency in addition to the exceptional optoelectronic properties of CsPbBr_3_ and GaAs.

### External quantum efficiency spectra

**Fig. 3:**
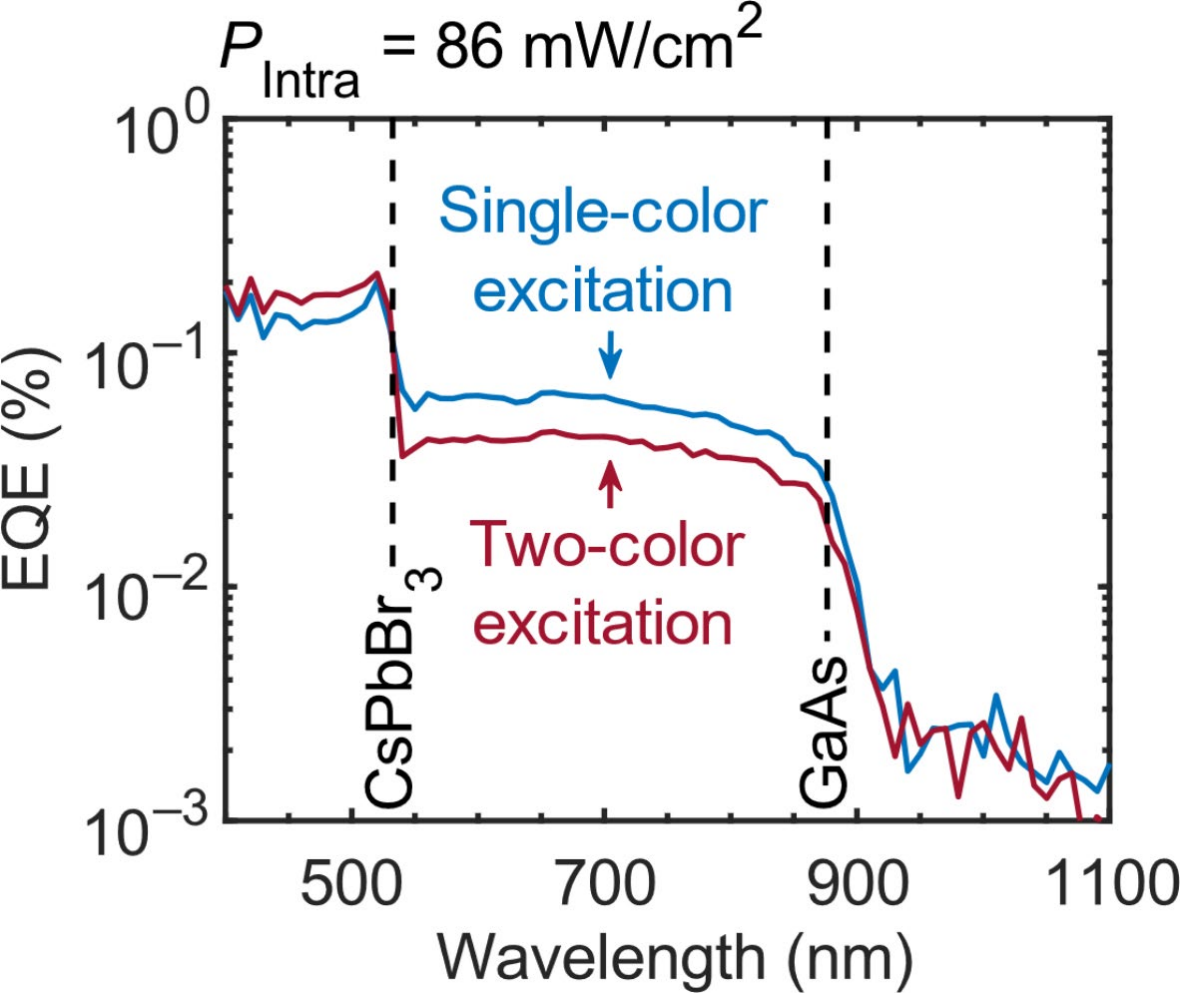
The EQE spectra of the CsPbBr_**3**_/GaAs-based TPU-SC. The spectra were obtained with and without illumination with IR photons (*P*_Intra_ = 86 mW/cm^2^) shown by the dark red and blue curves, respectively. The band edges of CsPbBr_3_ and GaAs are indicated by the vertical dashed lines.

The room-temperature external quantum efficiency (EQE) spectra of our CsPbBr_3_/GaAs-based TPU-SC are shown in Fig. 3. The blue curve represents the EQE spectrum measured under single-color excitation conditions, which means that carrier generation occurs mainly through interband excitation. The EQE signal gradually increases as the excitation wavelength decreases. At wavelengths longer than ~ 900 nm, there is no EQE signal, because these photons cannot excite any semiconductor in this device. The EQE signal shows a sharp onset when the excitation wavelength is about 900 nm, where GaAs starts to absorb the photons. The signal reaches a plateau at about 870 nm, which corresponds to the GaAs bandgap. As a result of the built-in electric field, the photogenerated electrons in the CB of GaAs (generated by the photons in the wavelength range from approximately 530 to 870 nm) drift toward HI-I, while the photogenerated holes drift to the rear electrode (the holes are not influenced by HI-I). The electrons are trapped at the interface states of HI-I and accumulate there. These accumulated electrons partially recombine with the holes that reach HI-I by diffusion. We described this scenario in previous publications^9–12^. A part of the electrons at HI-I can overcome the energy barrier by thermal excitation (evidence for thermionic emission is provided in Supplementary Information Section S2). At an excitation wavelength of about 530 nm, the EQE signal abruptly increases, because the corresponding photon energy is high enough to induce interband transitions in both CsPbBr_3_ and GaAs. As the wavelength becomes shorter, the photon energy becomes higher than the CsPbBr_3_ bandgap energy and the photons are stronger absorbed by the CsPbBr_3_ layer (the number of photons that reach the GaAs layer becomes smaller). Since the holes generated in the CsPbBr_3_ VB have a longer path to the rear electrode, which implies a larger recombination probability, the EQE should decrease if the excitation wavelength is reduced further.

The dark red curve in Fig. 3 represents the EQE spectrum measured under two-color excitation conditions (hereafter, *P*_Intra_ is always used to refer to the power density of the 1319-nm infrared (IR) laser beam). The comparison with the data for single-color excitation reveals that the EQE in the wavelength range between the CsPbBr_3_ bandgap and the GaAs bandgap (~ 530–870 nm) decreases when the device is additionally irradiated with the IR photons, and in the wavelength range below 530 nm (the CsPbBr_3_ bandgap), the IR photons lead to an enhancement of the EQE signal. In contrast, our previous work on TPU-SCs with a single heterointerface showed an IR-induced EQE enhancement in the range between the Al_0.3_Ga_0.7_As and GaAs, and a drastic reduction of the enhancement in the short-wavelength range. In particular, in an Al_0.3_Ga_0.7_As/GaAs-based TPU-SC, there is small IR-induced EQE enhancement when the wavelength of the photons for interband excitation is shorter than 680 nm (the Al_0.3_Ga_0.7_As bandgap). The small IR-induced EQE is significantly smaller than the EQE measured in the wavelength range between the Al_0.3_Ga_0.7_As bandgap and the GaAs bandgap (680–870 nm), i.e., the difference is approximately 50%^9^. This result is attributed to the fact that high-energy photons are strongly absorbed by the Al_0.3_Ga_0.7_As because of high absorption coefficients^9–12^. In addition, the phenomenon obeys Beer–Lambert law stating that the high-energy photons exhibit shallow penetration depths. Therefore, reducing photon wavelengths (increasing photon energy) causes a reduction in the number of photons that reach the GaAs layer. Hence, the electron density at the heterointerface is reduced because the electron density is only generated by the interband photoexcitation in the GaAs. As a result, the IR-induced EQE enhancement gradually decreases as the wavelength of the photons for interband excitation becomes shorter; for the ultimate case, if there are no photogenerated electrons in the GaAs CB, the IR laser beam has no effect, because the photogenerated electrons in the Al_0.3_Ga_0.7_As CB do not accumulate at the energy barrier^9–11^.

To clarify the behavior of our CsPbBr_3_/GaAs-based TPU-SC, we consider our previous results on a double-heterointerface TPU-SC with an additional Al_0.7_Ga_0.3_As (~ 2.05 eV) layer grown on the Al_0.3_Ga_0.7_As/GaAs (1.79 eV/1.42 eV) structure: this device exhibited an IR-induced EQE improvement in the short-wavelength range^12^. Hence, the IR-induced EQE enhancement observed in Fig. 3 at wavelengths below 530 nm is likely due to the interface states of HI-II. To support this interpretation, we estimated the fraction of 500-nm light absorbed in each layer of the CsPbBr_3_/GaAs-based TPU-SC (the calculation details are explained in Section S3 of the Supplementary Information). Our estimation reveals that photons with such short wavelengths do not induce electron accumulation at HI-I. Furthermore, in this measurement, non-linear phenomena such as multi-photon absorption in CsPbBr_3_ are negligible, because we used a tungsten-halogen lamp and a continuous-wave (CW) laser for the excitation, i.e., light sources with a relatively low energy density. Therefore, we believe that the IR-induced EQE enhancement in the short-wavelength regime is caused by electron accumulation at HI-II.

Conversely, it has been found that the EQE signal in the wavelength region between the bandgaps of CsPbBr_3_ and GaAs reduces with the IR irradiation. The change in EQE with the IR irradiation strongly depends on the excitation power density. We discuss the details in Excitation Power-Dependence of the Short-circuit Current.

**Fig. 4:**
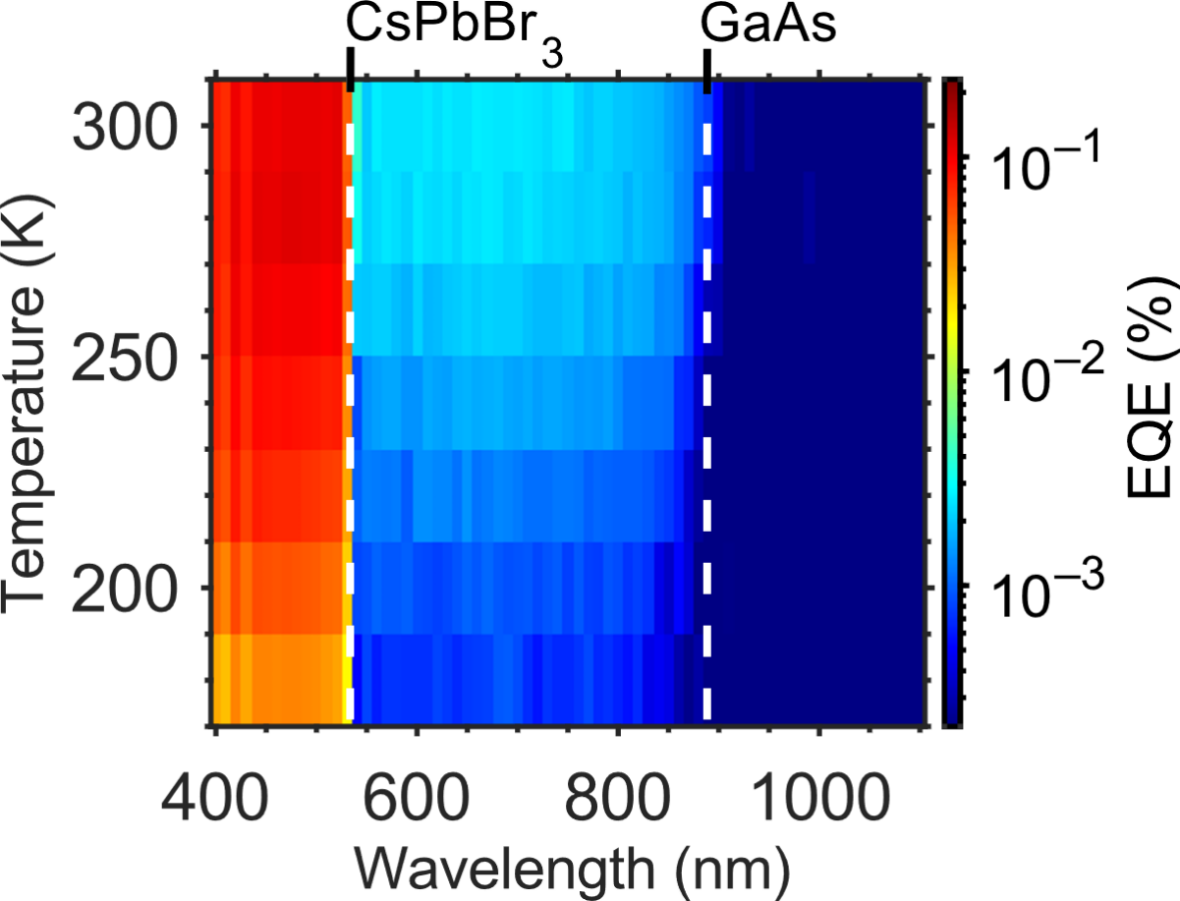
Temperature-dependence of the EQE of the CsPbBr_3_/GaAs-based TPU-SC for single-color excitation. The data reveals a clear bandgap shift of GaAs. The white dashed lines at 530 nm and 870 nm indicate the band edges of CsPbBr_3_ and GaAs at room temperature, respectively.

Figure 4 shows the temperature dependence of the EQE spectrum for single-color excitation (the wavelength of the photons for interband excitation is shown on the *x*-axis, and the temperature is shown on the *y*-axis). At temperatures below 180 K, the CsPbBr_3_/GaAs-based TPU-SC hardly produces photocurrent due to the CB discontinuity at HI-I: The difference between the electron affinities *χ* of CsPbBr_3_ and GaAs provides a barrier height of 0.77 eV at HI-I. Since the thermal activation energy at 297 K is only 0.026 eV, the electrons accumulated at the heterointerface can hardly overcome the barrier at much lower temperatures. In this measurement, the temperature governs the electronic excitation at HI-I, because 1319-nm (sub-bandgap) photons were not used. Consequently, Fig. 4 confirms the thermal activation of electrons accumulated at HI-I, which plays an important role in the photocurrent generation when sub-bandgap photons are absent^38^. Figure 4 also reveals a clear shift of the absorption edge of GaAs: it shows a blueshift with decreasing temperature due to the well-known effect responsible for the temperature-dependent changes in the bandgaps of III–V semiconductors^39,40^. The absorption edge of CsPbBr_3_, on the other hand, is almost insensitive to the temperature within the measured range.

It has been reported that the bandgap energy of lead bromide perovskites (*X*PbBr_3_, with *X* = FA^+^, MA^+^, or Cs^+^) decreases with decreasing temperature, in contrast to the behavior of III–V semiconductors. The increase of the *X*PbBr_3_ bandgap energy with increasing temperature is attributed to changes in the crystal phase. In general, *X*PbBr_3_ perovskites can exist in various crystal phases including tetragonal and cubic phases. In the case of CsPbBr_3_, the orthorhombic phase can be observed easily at room temperature, because of its good stability, and as the temperature increases, phase changes occur. Mannino et al. reported that the *E*_g_ of CsPbBr_3_ has a small dependence on the temperature in the range 200–300 K^28^. This phenomenon is caused by the thermal expansion of the orthorhombic phase. At about 380 K (107 °C), the CsPbBr_3_ bandgap changes abruptly due to the phase transition from the orthorhombic phase to the tetragonal phase (several investigations confirmed this phase transition already at 361 K^20,29,30^). This is in contrast to FAPbBr_3_ and MAPbBr_3_, where a tetragonal–cubic phase change occurs in the temperature range 200–300 K, and results in an *E*_*g*_ change of CsPbBr_3_ of approximately 0.02 eV in the range 200–380 K^28^. Within the temperature range used for Fig. 4, CsPbBr_3_ is in the orthorhombic phase. As a result, the absorption edge is almost constant 2.33 eV (the *E*_*g*_ change is within ~ 0.008 eV).

### Excitation power-dependence of the short-circuit current

**Fig. 5:**
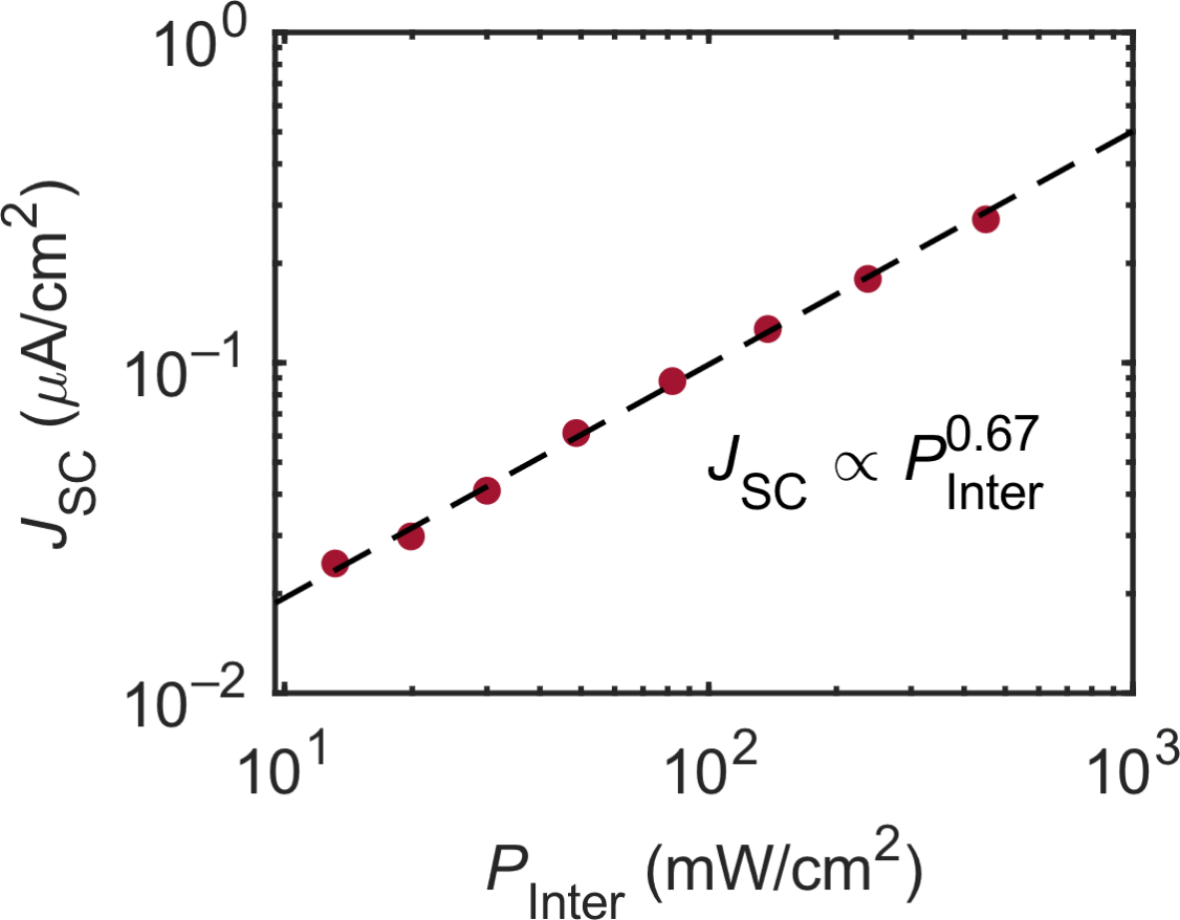
The *J*_SC_ for single-color excitation as a function of ***P***_Inter_.

Figure 5 shows the dependence of the short-circuit current (*J*_SC_) on 784-nm interband excitation power density (*P*_Inter_) for single-color excitation. The *J*_SC_ increases as *P*_Inter_ increases. This is the main feature of solar cells which can be supported by the *JV* characteristic curved in Fig. S4 of Supplementary Information. The 784-nm photons excite the GaAs substrate and intraband transitions are not induced, because sub-bandgap photons are absent. The photogenerated electrons in the CB of GaAs drift toward HI-I, where electrons accumulate due to the CB discontinuity. To overcome the barrier (0.77 eV), an energy is required that is nearly 30 times the thermal energy at room temperature (0.026 eV). Therefore, the temperature is important for the *J*_SC_ under single-color excitation conditions. The effect of the temperature is discussed in Supplementary Information Section S2.

To discuss the data in Fig. 5, we fitted the data to a single power law, ${\it\text{J}}_{{{\text{SC}}}} \propto {\it\text{P}}_{{{\text{Inter}}}}^{{{\it\text{ n}}}}$. The estimated power exponent is *n* = 0.67, which significantly deviates from unity. The reason for this sublinear power dependence is the weak built-in electric field and the recombination with holes: The electrons generated in the GaAs CB drift toward HI-I due to a weak electric field. As the electrons accumulate at HI-I, the electric field becomes weaker, which increases the electron–hole recombination rate. Therefore, the rate of thermal activation at HI-I decreases as *P*_Inter_ increases, leading to the observed sublinear relationship.

Since the energy difference of the CB at the HI-I is 30 times larger than thermal energy at room temperature, the assumption of two-photon absorption, i.e. one 784-nm photon excites an electron from the VB to the CB of GaAs and another one excites the electron in the CB of GaAs to the CB of CsPbBr_3_, is valid and possible. However, Fig. 5 shows the sublinear power dependence arising from the single-photon absorption. Furthermore, in our measurement of temperature-dependent photocurrent with Arrhenius fitting (see Supplementary Information Section S2), we used a constant intensity of 784-nm photons. Therefore, the effect of 784-nm photons on the intraband excitation is negligible because they are a controlled variable. Hence, we believe the observed photocurrent under the 784-nm photoexcitation is due to thermal activation at the CsPbBr_3_/GaAs heterointerface (HI-I) even high barrier height of 0.77 eV, not due to two-photon absorption.

**Fig. 6:**
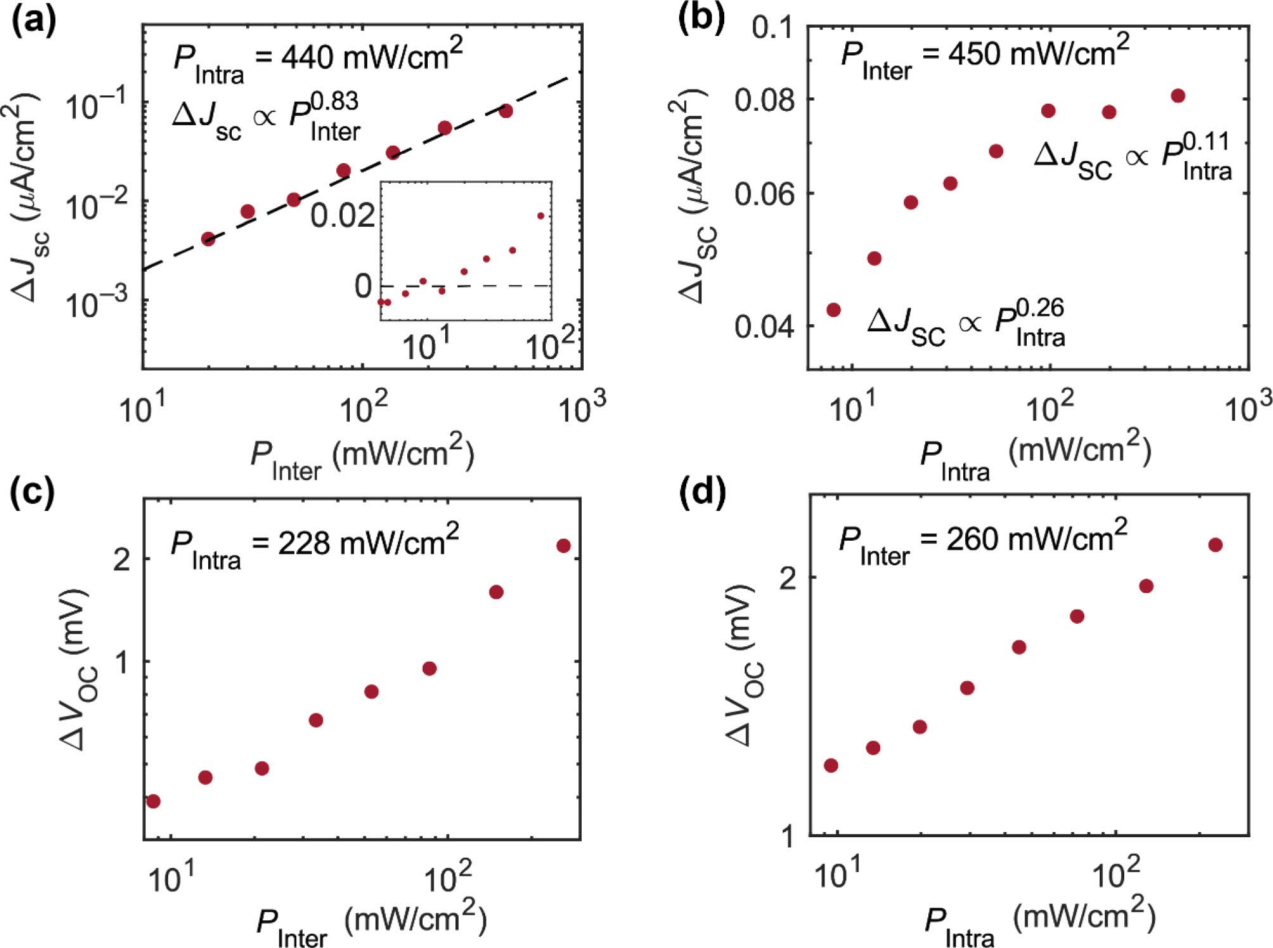
The IR-induced gain in the short-circuit current (Δ*J*_SC_) and the open-circuit voltage (Δ*V*_OC_) for single-color excitation as a function of*P*_Inter_and*P*_Intra_. (**a**) Δ*J*_SC_ as a function of *P*_Inter_ for a constant 1319-nm photon flux. (Inset) Linear plot for the vertical axis of Figure (**a**). The same data as in Figure (**a**) is used but is visualized from *P*_Inter_ = 4.2 mW/cm^2^ to *P*_Inter_ = 100 mW/cm^2^. The inset figure axes are identical to Figure (**a**). (**b**) Δ*J*_SC_ as a function of *P*_Intra_ for a constant 784-nm photon flux. (**c**) Δ*V*_OC_ as a function of *P*_Inter_ for a constant 1319-nm photon flux. (**d**) Δ*V*_OC_ as a function of *P*_Intra_ for a constant 784-nm photon flux.

Figure 6a shows the photocurrent enhancement under short-circuit conditions (Δ*J*_SC_ = *J*_SC, with infrared_ – *J*_SC, without infrared_) as a function of the power density of the 784-nm laser beam in the case of intraband excitation power density *P*_Intra_ = 440 mW/cm^2^. The photocurrent enhancement can be observed as well in the *JV* characteristic curve shown in Fig. S4 of Supplementary Information. The Δ*J*_SC_ increases with *P*_Inter_, because stronger interband excitation provides a higher electron density at HI-I (the 784-nm photons induce interband transitions in GaAs, and the photogenerated electrons are transported to HI-I). The data can be well fitted using a single power law, Δ*J*_SC _$\propto {\it\text{P}}_{\text{Inter}}^{\it\text{ n}}$, with *n* = 0.83. Ideally, Δ*J*_SC_ should be proportional to the electron density at HI-I. Furthermore, the recombination in ZnO and CsPbBr_3_ (as well as that at HI-II) should be negligible, because the 784-nm photons induce interband transitions only in the GaAs layer, and thus there are no photogenerated holes in ZnO and CsPbBr_3_. The sublinear characteristic, therefore, originates from a recombination within GaAs or at HI-I. While stronger interband excitation leads to a higher electron density at HI-I, this increased electron density reduces the built-in electric field, facilitating electron–hole recombination within GaAs. Hence, the electron density at HI-I sublinearly increases with *P*_Inter_. This behavior was also observed in our previous reports^10,41^. The inset figure shows the same data as Fig. 6a, which is the Δ*J*_SC_ as a function of 784-nm interband excitation power density, in semilogarithmic plot visualized from *P*_Inter_ = 4.2 mW/cm^2^ to *P*_Inter_ = 100 mW/cm^2^. The black dashed line marks zero point. Evidently, the Δ*J*_SC_ scatters, and is mostly negative, when the interband excitation power density is below ~ 20 mW/cm^2^. This corresponds to the 784-nm power density used to measure the EQE. The comparison between photocurrent and EQE enhancement is discussed next.

Figure 6b clarifies the dependence of Δ*J*_SC_ on *P*_Intra_ in the case of *P*_Inter_ = 450 mW/cm^2^. This data cannot be fitted to Δ*J*_SC _$\propto {\it\text{P}}_{\text{Intra}}^{\it\text{ n}}$, because the data shows a gradual change of the power exponent, i.e. the value of *n* decreases as *P*_Intra_ is increased. In the low excitation regime, we find *n* = 0.26, which is the maximum value in the measured range of power densities. The minimum value is *n* = 0.11, observed at *P*_Intra_ ~ 450 mW/cm^2^. This reduction of *n* can be interpreted as a reduction of the carrier separation efficiency at HI-I. Since the 784-nm photons induce interband transitions only in the GaAs layer, recombination in ZnO and CsPbBr_3_ (including that at HI-II) can be ignored. Therefore, the observed reduction of the carrier separation efficiency occurs at HI-I. The origin of the reduction is a lower built-in electric field at higher electron densities in the CBs of ZnO and CsPbBr_3_ (and at HI-II), because the electrons that are optically excited to the CB of CsPbBr_3_ hardly drift to HI-II if the built-in field is weak. This means that the functionality of HI-I is limited by the electron densities in ZnO and CsPbBr_3_^12^.

**Fig. 7:**
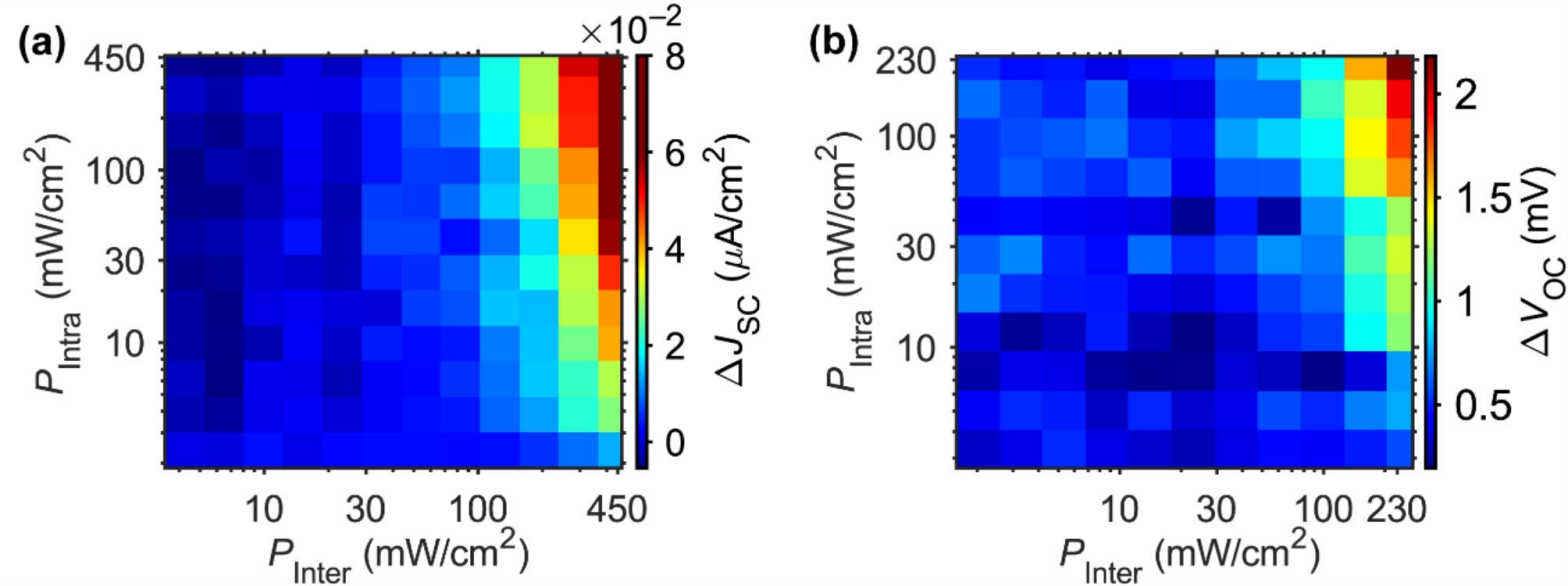
Visualization maps depicting overall tendencies (**a**) The IR-induced gain in the short-circuit current (Δ*J*_SC_) and (**b**) The open-circuit voltage (Δ*V*_OC_) as functions of *P*_Inter_ and *P*_Intra_.

Although the Δ*J*_SC_ data at first seems inconsistent with the EQE results, both results are actually in agreement. To understand this, we need to consider that a tungsten-halogen lamp was employed for the EQE measurement, which only provided 6.0 × 10^–4^ mW/cm^2^ at about 780 nm (a photon flux of 2.4 × 10^16^ m^–2^s^–1^). As shown in the inset figure of Fig. 6a, intraband transitions hardly occur as long as *P*_Inter_ is below 20 mW/cm^2^ (a photon flux of 8.0 × 10^20^ m^–2^s^–1^). Therefore, the power density of the monochromatic beam for interband excitation used in the EQE measurement was too low. For the Δ*J*_SC_ measurements as a function of *P*_Inter_ (Fig. 6a), we employed a CW solid-state laser operating at 784 nm, which provided a photon flux ranging from 8.0 × 10^20^ m^–2^s^–1^ to 2.0 × 10^22^ m^–2^s^–1^ for interband excitation. Similarly, the photon flux for interband excitation, used in the Δ*J*_SC_ measurements as a function of *P*_Intra_ (Fig. 6b), was 1.8 × 10^22^ m^–2^s^–1^. Therefore, the positive Δ*J*_SC_ can be observed. Additionally, Fig. 7a, in Overall Tendencies of Δ*J*_SC_ and Δ*V*_OC_, provides an overall image of the Δ*J*_SC_ as functions of 784-nm interband and 1319-nm intraband excitation power densities.

The mechanisms playing roles in the EQE reduction or the scattered Δ*J*_SC_ are uncertain. However, a possible scenario would be due to the photoactivated trap states within CsPbBr_3_ perovskite or at heterointerfaces. It is reported that near-infrared photons can cause reversible crystal decomposition under irradiation of low x-ray flux^42^. This changes the binding energy of Pb 4*f* which may cause defect states within the perovskite as well as its interfaces. Furthermore, several publications have proposed self-absorption-induced trap-state activation since long free exciton lifetimes have been observed. The self-absorption-induced trap-state activation is also used to explain the blinking photoluminescent emission of CsPbBr_3_, i.e. the blinking photoluminescence is caused by the localized states in CsPbBr_3_ and at its surface or interface^43–45^. Therefore, we interpret the scattered Δ*J*_SC_ as follows: weak interband excitation intensity, with wavelengths ranging from ~ 530–870 nm, produces small photogenerated carrier density in GaAs. The photogenerated electrons drift toward the HI-I while the holes drift toward the bottom electrodes. Since interface states exist at the HI-I, the electrons need to deactivate the states in order not to recombine non-radiatively with partial holes that can accumulate at the HI-I by a diffusion process. The electrons can be thermally excited and be collected at the top electrodes as photocurrent, and hence, the EQE signals. With additional 1319-nm photoexcitation, the interface states and localized states within CsPbBr_3_ can be activated. This may result in an increase in non-radiative recombination which lessens the photocurrent. Therefore, the EQE decreases with the presence of 1319-nm photons compared to the EQE measured without additional 1319-nm photoexcitation. With increasing 784-nm photons exciting GaAs, photogenerated electrons increase, as do the accumulated electrons at the HI-I. The electrons deactivate the photon-induced trap states by occupying the states. However, the photogenerated electron density is sufficiently high to be transported and collected at the top electrodes. Hence, the photocurrent is enhanced by 1319-nm photoexcitation when the 784-nm photon intensity is high and the EQE can be enhanced.

### Excitation power-dependence of the open-circuit voltage

Figure 6c shows the IR-induced gain in the open-circuit voltage (Δ*V*_OC_ = *V*_OC, with infrared_ – *V*_OC, without infrared_) as a function of *P*_Inter_ in the case of *P*_Intra_ = 228 mW/cm^2^, and Fig. 6d shows the Δ*V*_OC_ as a function of *P*_Intra_ in the case of *P*_Inter_ = 260 mW/cm^2^. The positive Δ*V*_OC_ values originate from the increase of the electron density in the CB of CsPbBr_3_ caused by the additional illumination with the 1319-nm photons (optically induced intraband transitions).

Figure 6c shows an increase in Δ*V*_OC_ with increasing *P*_Inter_. We consider that the carrier extraction at HI-I is an adiabatic optical process^9,38^. Therefore, the *V*_OC_ increases. Compared to the thermionic emission and tunneling processes at HI-I, the intraband transitions induced by the 1319-nm photons provide an increased electron population in the CB of CsPbBr_3_. This induces a split between electron quasi-Fermi levels of CsPbBr_3_ and GaAs. The positive Δ*V*_OC_ values in Fig. 6c and 6d evidence the presence of an adiabatic TPU process at HI-I. Since stronger interband excitation causes a higher electron density at HI-I, the Δ*V*_OC_ increases with *P*_Inter_. In addition, we observed increasing *V*_OC_ with increasing *P*_Inter_ shown in Fig. S5 of Supplementary Information.

Similarly, Fig. 6d shows an increase of Δ*V*_OC_ with increasing *P*_Intra_, in agreement with the Δ*J*_SC_ data shown in Fig. 6b. As *P*_Intra_ increases, more electrons accumulated at HI-I are upconverted to the CB of CsPbBr_3_, which induces a widening of the quasi-Fermi-level splitting.

### Overall tendencies of Δ*J*_SC_ and Δ*V*_OC_

Figure 7a shows Δ*J*_SC_ as functions of *P*_Inter_ (*x*-axis) and *P*_Intra_ (*y*-axis). The data scatters around zero and mostly shows negative values when *P*_Inter_ is below ~ 20 mW/cm^2^. When *P*_Inter_ exceeds 20 mW/cm^2^, an increase in *P*_Inter_ also leads to a remarkable increase in Δ*J*_SC_. Note that, in the region below 20 mW/cm^2^, the data is independent of the intensity of the IR light. We interpret this behavior as follows: Since the electron density in the GaAs CB is mainly determined by the intensity of the photons used to induce interband transitions, the electron density remains low as long as *P*_Inter_ is low. In addition, we used a *p*-GaAs substrate for the NGS layer (there is no *i*-GaAs layer), which makes it more difficult to increase the electron density compared to an Al_0.3_Ga_0.7_As/GaAs-based TPU-SC. Because the possibility of upconversion at HI-I is determined by the electron density at the initial energy levels for the intraband transitions, there is almost no Δ*J*_SC_ in the case of weak interband excitation, even at high values of *P*_Intra_.

The result of the negative Δ*J*_SC_ when *P*_Inter_ is below ~ 20 mW/cm^2^, shown in Fig. 7a, agrees with the EQE results in Fig. 3. Since the photons used for interband excitation in our EQE measurement were generated by a tungsten-halogen lamp, the photon density was below the threshold. The power density of the monochromatic 780-nm beam generated by the tungsten-halogen lamp and the monochromator was approximately 6.0 × 10^–4^ mW/cm^2^, which is equivalent to 0.005 suns. In other words, the used power density was too small to produce a significant density of electrons at HI-I. According to Fig. 7a, *P*_Inter_ needs to be higher than ~ 20 mW/cm^2^ to let a significant amount of electrons accumulate at HI-I. This result points out that electron accumulation at the heterointerface is a prerequisite for adiabatic intraband transitions caused by sub-bandgap photons. It is well known that non-radiative recombination plays a significant role due to the presence of interface states^46,47^. For two-color excitation conditions, the IR light is able to activate the interface states^42–45,48^, which additionally induces non-radiative recombination at the heterointerface, and, thereby, the IR-induced EQE reduction occurs. The detailed mechanisms were elucidated in Excitation Power-Dependence of the Short-circuit Current.

Figure 7b shows Δ*V*_OC_ as functions of *P*_Inter_ and *P*_Intra_. Overall, this map shows an increase in Δ*V*_OC_ as both *P*_Inter_ and *P*_Intra_ increase. Furthermore, a feature identical to that observed in Fig. 7a can be seen: when *P*_Inter_ is below ~ 20 mW/cm^2^, the Δ*V*_OC_ values are scattered around zero. The increase with either *P*_Inter_ or *P*_Intra_ only appears for sufficiently high *P*_Inter_ values. This feature indicates that the mechanism responsible for the IR-induced enhancement of *V*_OC_ is identical to that for the IR-induced enhancement of *J*_SC_; the adiabatic upconversion of electrons contributes to both photocurrent and photovoltage. Furthermore, also the threshold characteristic in Fig. 7b is due to the requirement of a sufficiently high electron density at HI-I.

### Influence of the temperature

In this section, the effect of the temperature on the photocurrent and photovoltage of the CsPbBr_3_/GaAs-based TPU-SC is discussed. The temperature dependence of *J*_SC_ for single-color excitation with *P*_Inter_ = 428 mW/cm^2^ is shown in the Arrhenius plot in Fig. S2 of Supplementary Information. In general, the *J*_SC_ increases with temperature, in agreement with the temperature dependence of the EQE in Fig. 4. This increase evidences thermionic emission at least at one of the two heterointerfaces in this device, since a higher temperature implies a higher thermal energy. Hence, at higher temperatures, the photogenerated electrons in the CB of GaAs can more easily overcome the band discontinuity by thermal excitation and then are collected at the front electrode.

**Fig. 8:**
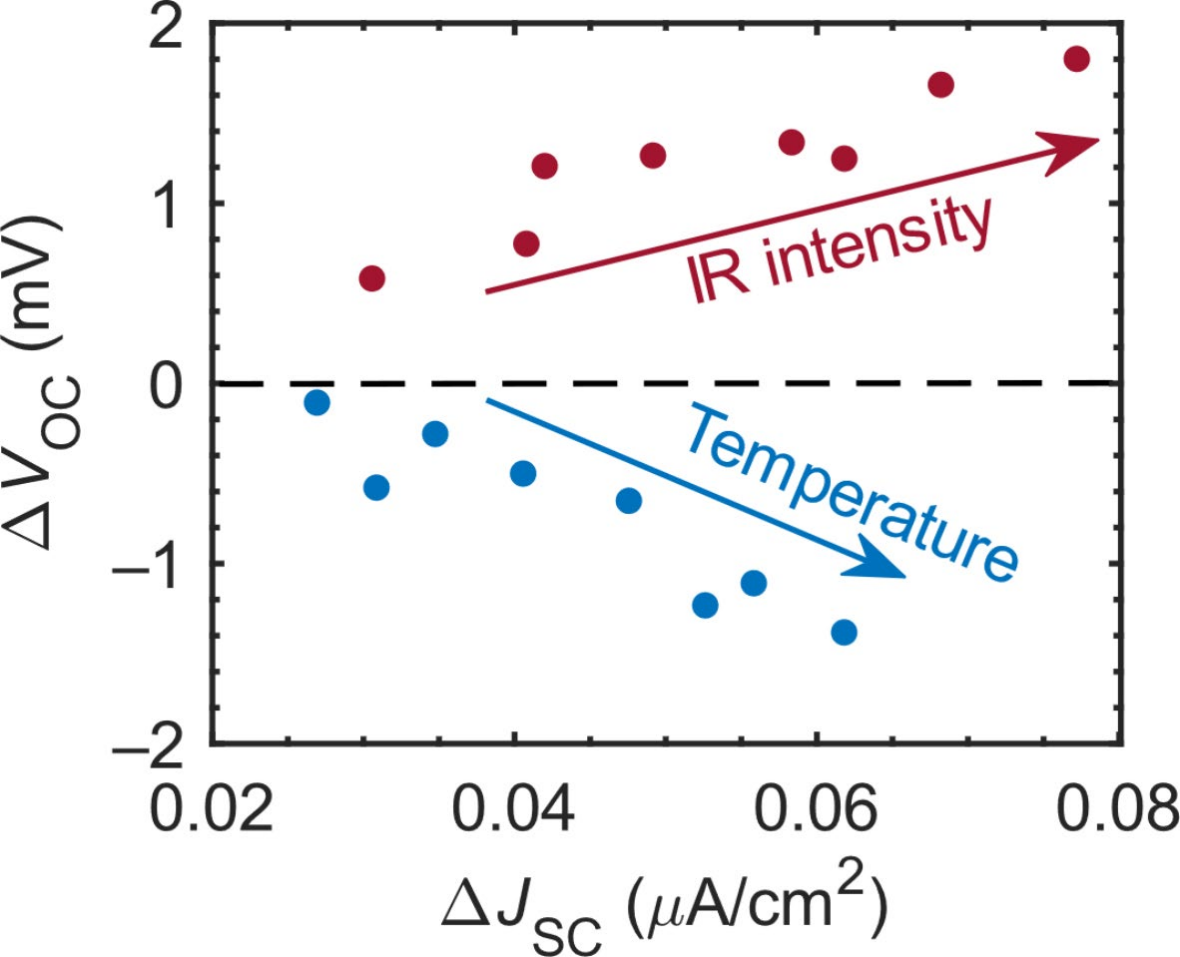
Comparison of Δ*J*_SC_–Δ*V*_OC_data sets. The data was obtained either at room temperature as a function of *P*_Intra_ (the dark red points) or as a function of the temperature for *P*_Intra_ = 0 (the blue points).

As shown in Fig. 7, Δ*J*_SC_ and Δ*V*_OC_ increase with both *P*_Inter_ and *P*_Intra_ (if *P*_Inter_ > 20 mW/cm^2^). In Fig. 8, we provide additional evidence for the presence of optically induced intraband transitions at HI-I. Figure 8 shows two data sets of Δ*V*_OC_ as a function of Δ*J*_SC_ for *P*_Inter_ = 450 mW/cm^2^: The dark red points represent the Δ*J*_SC_–Δ*V*_OC_ data acquired at room temperature as a function of *P*_Intra_ (the data showing the IR-induced gain in *J*_SC_ and *V*_OC_). This dataset indicates an increase in both Δ*J*_SC_ and Δ*V*_OC_ with increasing *P*_Intra_. The blue points represent the Δ*J*_SC_–Δ*V*_OC_ data for *P*_Intra_ = 0 as a function of the temperature (the data showing the temperature-induced changes in *J*_SC_ and *V*_OC_). This dataset exhibits a clearly different feature, i.e., the Δ*V*_OC_ decreases as Δ*J*_SC_ increases. These two data sets allow us to compare the influence of optically induced intraband transitions (sub-bandgap photons) and thermal activation (thermal energy) on the SC performance.

In Fig. S2 of Supplementary Information, we demonstrated thermal activation, which provides more additional photocurrent (Δ*J*_SC_) as the temperature increases. However, the advantage gained by this process is not identical to the advantage gained by the photocurrent increase through optically induced intraband transitions. To understand the difference, the Δ*V*_OC_ in the case of thermal activation needs to be considered. It is well-known that the *V*_OC_ has a strong inverse dependence on the dark saturation current, which increases with temperature^49,50^ (see Section S12 of Supplementary Information). Therefore, the *V*_OC_ decreases with increasing temperature and the temperature-induced change in Δ*V*_OC_ becomes more negative with increasing temperature (Fig. 8; blue data), in contrast to the Δ*V*_OC_ due to the IR-induced intraband transitions (Fig. 8; dark red data). Because IR-induced intraband transitions are optical processes, the Δ*V*_OC_ is positive (the quasi-Fermi-level splitting increases as discussed in Excitation Power-Dependence of the Open-Circuit Voltage). Consequently, the two Δ*J*_SC_–Δ*V*_OC_ data sets have a different origin for the observed Δ*J*_SC_, and the positive Δ*V*_OC_ values obtained using sub-bandgap photons confirm the presence of adiabatic optical excitation at HI-I^9,38^.

## Conclusions

We have presented current and voltage data of a CsPbBr_3_/GaAs-based TPU-SC under different excitation conditions. The EQE measured with and without additional illumination with sub-bandgap photons revealed clear absorption edges corresponding to the semiconductors in this SC. The enhancement of the photocurrent and photovoltage observed in the case of additional illumination with sub-bandgap photons confirms a TPU process at the CsPbBr_3_/GaAs heterointerface. This distinguishes the effect of optically induced intraband transitions from the effect of elevated temperatures. We elucidated the carrier dynamics in the SC by investigating the influences of the temperature as well as the power densities of the two laser beams used to induce interband and intraband transitions. Despite the observation of a TPU process in this SC, the efficiency of this SC is still too low for applications. The *J*_SC_, *V*_OC_, and fill factor measured under 1-sun illumination are indicated in Section S6 of Supplementary Information. The optimization of the CsPbBr_3_ quality may lead to better results. Furthermore, the large difference between the electron affinities of CsPbBr_3_ and ZnO might have led to an open-circuit voltage reduction^51^. To overcome this problem, it has been proposed to use AlGaN as the ETL^52^. The TPU process observed at the CsPbBr_3_/GaAs heterointerface in this work is a first step in studying perovskite/III–V semiconductor interfaces. We believe that the observation of the TPU process at the CsPbBr_3_/GaAs heterointerface is important for the future development of TPU-SCs, and hence, high-efficiency single junction SCs.

## Methods

### Fabrication of CsPbBr_3_/GaAs-based TPU-SCs

We fabricated the TPU-SC as follows: First, a 1-inch *p*-doped GaAs substrate (001) was washed in acetone, methanol, isopropanol, and deionized water (in each step, sonication was performed for 15 min, and the whole cleaning process is referred to as the AMID process). Then, we deposited Au-Zn/Au electrodes at the rear surface of the GaAs substrate. The deposition of Au-Zn/Au was done using a thermal evaporator. We deposited about 200 nm of Au-Zn, and this was followed by 500 nm of Au. To improve the ohmic characteristics of the *p*-GaAs/Au-Zn/Au contact, we annealed the substrate at 430 °C for 2.5 min under N_2_ gas. The substrate was washed again using the AMID process. Then, the substrate was treated with hydrofluoric acid for 20 s to remove the native oxide layer on the surface. After that, the substrate was cleaned in a UV/O_3_ cleaner for 10 min.

We chose a conventional multi-step spin-coating deposition technique to grow the $\:\text{CsPbB}{\text{r}}_{\text{3}}$ layer, since this allows us to easily prepare a perovskite layer with high crystallinity and good surface coverage under ambient conditions^18,20,53^. We prepared two precursor solutions for the $\:\text{CsPbB}{\text{r}}_{\text{3}}$ layer: (A) 1.00 M of lead (II) bromide (PbBr_2_, Tokyo Chemicals Industry) was dissolved in *N*,* N*-Dimethylformamide (DMF, Sigma-Aldrich), and (B) 0.07 M of cesium bromide (CsBr, Tokyo Chemicals Industry) was dissolved in a mixture of methanol (FUJIFILM Wako Pure Chemical Corp.) and deionized water with a ratio of 5:1. Both precursor solutions were stirred at 90 °C for 30 min until the solution became clear, and then both solutions were filtered using microporous filters to remove microparticles and microcrystals. The precursor solution A was drop-casted on the cleaned *p*-GaAs substrate until the surface was covered with the solution. Then, the substrate was spun at 2000 rpm for 30 s. Five seconds after the start of the spin-coating process, 100 µL of chlorobenzene (FUJIFILM Wako Pure Chemical Corp.) was dropped on the spinning substrate. Subsequently, the substrate was heated at 90 °C for 30 min under ambient conditions. Then, precursor solution B was drop-casted on the substrate. The substrate was again spun at 2000 rpm for 30 s, and then heated at 250 °C for 5 min. As a result of the optimization of the number of deposition cycles of precursor B, we repeated this process four times to achieve a high-purity CsPbBr_3_ perovskite layer and to suppress the formation of CsPb_2_Br_5_ and Cs_4_PbBr_6_ (it has been reported that the formation of the latter two phases depends on the concentration of PbBr_2_ and CsBr^20^, and we optimized the concentration of CsBr by using several the deposition cycles). The high purity of the CsPbBr_3_ perovskite phase can be confirmed in the X-ray diffractograms shown in Section S8 of the Supplementary Information (the optimization of CsBr deposition cycles was done using glass substrates). We also confirmed the formation of the CsPbBr_3_ perovskite phase by photoluminescence (PL) spectroscopy. The PL spectrum in Section S9 the Supplementary Information shows a PL peak at about 535 nm, which coincides with the peak position reported in previous publications^18,20,22,37^.

The ZnO ETL was deposited on the CsPbBr_3_/*p*-GaAs substrate by sputtering. The ZnO target was purchased from Furuuchi Chemical. The background pressure inside the chamber was at 7.3 × 10^− 4^ Pa, and for the sputtering process we first supplied a mixture of Ar and $\:{\text{O}}_{\text{2}}$ (with a 1:1 ratio) to the chamber until the total pressure reached 4.5 to 5 Pa. Then, an Ar: O_2_ plasma was generated by applying an electric power of 50 W and we gradually increased the applied power to 100 W while the Ar:O_2_ pressure was gradually reduced to 1.3 Pa. After the pressure reached 1.3 Pa, the deposition was continued for 5 min. With this procedure, an approximately 130-nm-thick ZnO ETL is obtained.

To finalize the TPU-SC device, a 450-nm-thick Ag electrode was deposited on the top of the ZnO layer. The electrode was patterned using a metal mask with an aperture size of 3.0 mm × 3.0 mm.

Regarding the device stability, we observed that the stability can be up to 9–10 days approximately, i.e. EQE, *J*_SC_, and *V*_OC_, including their enhancement with additional 1319-nm photoexcitation can be properly observed. However, the CsPbBr_3_ films show incredible stability. Section S7 of Supplementary Information shows the absorption spectrum of four-month left CsPbBr_3_ films. The absorption edge clearly appears. Since the device stability is not good, the experiments, discussed in this article, were measured within 3–5 days in order to avoid the stability problems.

### EQE measurements

To measure the EQE, we employed a tungsten-halogen lamp as a light source for interband excitation. This lamp provides a broad spectrum, and the wavelength-integrated power density was 0.5 mW/cm^2^, which is much smaller than the solar irradiance. The lamp was combined with a 140-mm single monochromator to select specific wavelengths for excitation (note that the output beam intensity depends on the wavelength). Furthermore, an optical chopper with a chopping frequency of 250 Hz was inserted into the excitation path. This monochromatic light was used to induce interband transitions (the beam spot diameter on the SC surface was 1.2 mm). The short-circuit current was amplified by a current amplifier and detected by a lock-in amplifier synchronized to the optical chopper. Moreover, a fixed fraction of the excitation light was detected by a Si photodetector to estimate the number of incident photons at a given wavelength. This setup is for the EQE measurements under single-color excitation conditions, which were performed at room temperature without a temperature controller.

For the EQE measurements under two-color excitation conditions, we additionally illuminated the device with 1319-nm photons generated by a CW solid-state laser. These IR photons can induce intraband transitions in addition to the interband transitions induced by the light from the tungsten-halogen lamp. The power density of the IR laser beam was set to *P*_Intra_ = 86 mW/cm^2^ by a variable neutral-density filter. The beam spot diameter on the SC surface was 1.2 mm, and the excitation spot coincided with that for interband excitation, since we employed a single optical fiber to guide the excitation light to the sample.

To determine the temperature-dependence of the EQE spectrum, we used the same experimental setup as for the single-color excitation, but here the SC was installed in a cryostat to control the temperature. The EQE signals were recorded without the IR light in order to observe the effect of the temperature. The measurements were performed in the temperature range 180–300 K, because the photocurrent generated by the SC at temperatures lower than 180 K can hardly be detected.

### Excitation power-dependence of Δ*J*_SC_ and Δ*V*_OC_

The Δ*J*_SC_ and the Δ*V*_OC_ are important parameters for TPU-SCs. They are defined as Δ*J*_SC_ = *J*_SC, with infrared_ – *J*_SC, without infrared_ and Δ*V*_OC_ = *V*_OC, with infrared_ – *V*_OC, without infrared_. The Keithley 2400 source measure unit was employed to record the short-circuit current (*J*_SC_). First, we measured the *J*_SC_ for single-color excitation as a function of *P*_Inter_. Then, we measured the *J*_SC_ values under two-color excitation conditions. This allows us to determine Δ*J*_SC_ as functions of both *P*_Inter_ and *P*_Intra_. The intensities of the laser beams were controlled by two variable neutral-density filters. The values of Δ*V*_OC_ were obtained by measuring the *V*_OC_ values under single- and two-color excitation conditions, but the measurement of *V*_OC_ is slightly different from that of *J*_SC_. In the case of the *J*_SC_ measurement, the data was directly recorded by the source measure unit. On the other hand, the *V*_OC_ was determined by the following procedure: First, we applied a voltage where the current is approximately zero. Then, we used the *JV* data to estimate the values of *V*_OC_ using a linear least-squares method to estimate linear functions defined by the slope and the intercept on the *y*-axis. By obtaining these two parameters for each measured *JV* dataset, we can estimate the *V*_OC_ values, which are equal to the intercepts of the linear functions on the *x*-axis. As a result, the Δ*V*_OC_ can be estimated as functions of *P*_Inter_ and *P*_Intra_. Both types of measurements were conducted at room temperature without using a temperature controller.

We measured the temperature-induced changes in *J*_SC_ and *V*_OC_ under single-color excitation conditions. The same experimental system as for the temperature dependence of *J*_SC_ was employed (see Section S2 of Supplementary Information), and *P*_Inter_ was 450 mW/cm^2^. Note that for this experiment (which is discussed in Influence of Temperature), the parameters Δ*J*_SC_ and Δ*V*_OC_ are defined as Δ*J*_SC_ = *J*_SC, at high temperature_ – *J*_SC, at reference temperature_ and Δ*V*_OC_ = *V*_OC, at high temperature_ – *V*_OC, at reference temperature_ (the reference temperature is 282 K). Therefore, in this experiment, Δ*J*_SC_ and Δ*V*_OC_ express the changes in the photocurrent and the open-circuit voltage excluding the effect of the IR light (the TPU process).

## Electronic supplementary material

Below is the link to the electronic supplementary material.

Supplementary Material 1

## Data Availability

The data that support the findings of this study are available from the corresponding author upon reasonable request.

## References

[1] Kita, T., Harada, Y. & Asahi, S. *Energy Conversion Efficiency of Solar Cells*. 10.1007/978-981-13-9089-0 (2019).

[2] Shockley, W. & Queisser, H. J. Detailed balance limit of efficiency of *p-n* junction solar cells. *J. Appl. Phys.* **32**, 510–519 (1961).

[3] Hirst, L. C. & Ekins-Daukes, N. J. Fundamental losses in solar cells. *Prog. Photovoltaics Res. Appl.* **19**, 286–293 (2011).

[4] Henry, C. H. Limiting efficiencies of ideal single and multiple energy gap terrestrial solar cells. *J. Appl. Phys.* **51**, 4494–4500 (1980).

[5] Vos, A. & De Detailed balance limit of the efficiency of tandem solar cells. *J. Phys. D Appl. Phys.* **13**, 839–846 (1980).

[6] Hirst, L. C., Walters, R. J. & Führer, M. F. & Ekins-Daukes, N. J. Experimental demonstration of hot-carrier photo-current in an InGaAs quantum well solar cell. *Appl. Phys. Lett.* **104** (2014).

[7] Farrell, D. J., Sodabanlu, H., Wang, Y., Sugiyama, M. & Okada, Y. A hot-electron thermophotonic solar cell demonstrated by thermal up-conversion of sub-bandgap photons. *Nat. Commun.* **6**, 8685 (2015). 26541415 10.1038/ncomms9685 PMC4659835

[8] Luque, A. & Martí, A. Increasing the efficiency of Ideal Solar cells by photon induced transitions at Intermediate levels. *Phys. Rev. Lett.* **78**, 5014–5017 (1997).

[9] Asahi, S., Teranishi, H., Kusaki, K., Kaizu, T. & Kita, T. Two-step photon up-conversion solar cells. *Nat. Commun.* **8**, 14962 (2017). 28382945 10.1038/ncomms14962 PMC5384214

[10] Watanabe, K., Asahi, S., Zhu, Y. & Kita, T. Voltage boost effects in two-step photon upconversion solar cells with a modulation-doped structure. *J. Appl. Phys.* **130** (2021).

[11] Kinugawa, N., Asahi, S. & Kita, T. Reciprocal relation between intraband Carrier Generation and Interband recombination at the Heterointerface of two-step photon up-conversion solar cells. *Phys. Rev. Appl.* **14**, 14010 (2020).

[12] Mahamu, H., Asahi, S. & Kita, T. Multi-step photon upconversion in quantum-dot-based solar cells with a double-heterointerface structure. *J. Appl. Phys.* **133** (2023).

[13] Luque, A. et al. Absorption coefficient for the intraband transitions in quantum dot materials. *Prog. Photovoltaics Res. Appl.* **21**, 658–667 (2013).

[14] Kita, T., Maeda, T. & Harada, Y. Carrier dynamics of the intermediate state in InAs/GaAs quantum dots coupled in a photonic cavity under two-photon excitation. *Phys. Rev. B* **86**, 35301 (2012).

[15] Harada, Y., Maeda, T. & Kita, T. Intraband carrier dynamics in InAs/GaAs quantum dots stimulated by bound-to-continuum excitation. *J. Appl. Phys.* **113**, (2013).

[16] Asahi, S., Kusaki, K., Harada, Y. & Kita, T. Increasing conversion efficiency of two-step photon up-conversion solar cell with a voltage booster hetero-interface. *Sci. Rep.* **8**, 1–8 (2018). 29343735 10.1038/s41598-018-19155-x PMC5772604

[17] Hosokawa, H. et al. Solution-processed intermediate-band solar cells with lead sulfide quantum dots and lead halide perovskites. *Nat. Commun.* **10**, 43 (2019). 30626874 10.1038/s41467-018-07655-3 PMC6327045

[18] Gao, B. & Meng, J. High efficiently CsPbBr_3_ perovskite solar cells fabricated by multi-step spin coating method. *Sol. Energy* **211**, 1223–1229 (2020).

[19] Heo, J. H. et al. Planar CH _3_ NH _3_ PbI _3_ Perovskite solar cells with constant 17.2% average power conversion efficiency irrespective of the scan rate. *Adv. Mater.* **27**, 3424–3430 (2015). 25914242 10.1002/adma.201500048

[20] Ullah, S. et al. All-inorganic CsPbBr_3_ perovskite: a promising choice for photovoltaics. *Mater. Adv.* **2**, 646–683 (2021).

[21] Hu, Z. et al. Robust cesium lead Halide Perovskite Microcubes for frequency Upconversion Lasing. *Adv. Opt. Mater.* **5** (2017).

[22] Yuan, H. et al. All-inorganic CsPbBr_3_ perovskite solar cell with 10.26% efficiency by spectra engineering. *J. Mater. Chem. Mater.* **6**, 24324–24329 (2018).

[23] Zhao, Q. et al. Achieving efficient inverted planar perovskite solar cells with nondoped PTAA as a hole transport layer. *Org. Electron.* **71**, 106–112 (2019).

[24] Stoumpos, C. C., Malliakas, C. D. & Kanatzidis, M. G. Semiconducting tin and lead iodide perovskites with Organic cations: phase transitions, high mobilities, and Near-Infrared Photoluminescent Properties. *Inorg. Chem.* **2** (2013). 10.1021/ic401215x 23834108

[25] Zhang, J., Hodes, G., Jin, Z. & Liu, S. All-inorganic CsPbX_3_ Perovskite solar cells: progress and prospects. *Angew. Chem. Int. Ed.* **58**, 15596–15618 (2019). 10.1002/anie.201901081 30861267

[26] Song, J. et al. Ultralarge All-Inorganic Perovskite Bulk single crystal for high-performance visible–infrared dual-modal photodetectors. *Adv. Opt. Mater.* **5**, 1–8 (2017).

[27] Zeng, Q. et al. Inorganic CsPbI_2_Br Perovskite solar cells: the progress and perspective. *Solar RRL* **3**, 1–17 (2019).

[28] Mannino, G. et al. Temperature-dependent Optical Band gap in CsPbBr_3_, MAPbBr_3_, and FAPbBr_3_ single crystals. *J. Phys. Chem. Lett.* **11**, 2490–2496 (2020). 32148047 10.1021/acs.jpclett.0c00295 PMC7467746

[29] Stoumpos, C. C. et al. Crystal growth of the perovskite semiconductor CsPbBr_3_: a new material for high-energy radiation detection. *Cryst. Growth Des.* **13**, 2722–2727 (2013).

[30] Sutton, R. J. et al. Bandgap-tunable cesium lead Halide Perovskites with high thermal stability for efficient solar cells. *Adv. Energy Mater.* **6**, 1–6 (2016).

[31] Ezzeldien, M. et al. Electronic and optical properties of bulk and surface of CsPbBr_3_ inorganic halide perovskite a first principles DFT 1/2 approach. *Sci. Rep.* **11** (2021). 10.1038/s41598-021-99551-y PMC8523715 34663843

[32] Xia, Y. et al. Unexpected bowing band evolution in an all-inorganic CsSn_1 – X_Pb_x_Br_3_ perovskite. *RSC Adv.* **10**, 26407–26413 (2020). 35519736 10.1039/d0ra03709e PMC9055386

[33] Brennan, M. C. et al. Origin of the size-dependent Stokes Shift in CsPbBr_3_ perovskite nanocrystals. *J. Am. Chem. Soc.* **139**, 12201–12208 (2017). 28772067 10.1021/jacs.7b05683

[34] Mukherjee, S., Chakraborty, R., Paul, G. & Pal, A. J. Influence of self-doping on band-edges and Fermi energy of CsPbBr_3_. *Sol. Energy Mater. Sol. Cells* **248** (2022).

[35] Saxena, A. K. The conduction band structure and deep levels in Ga_1 – x_Al_x_As alloys from a high-pressure experiment. *J. Phys. C Solid State Phys.* **13**, 4323–4334 (1980).

[36] Bentell, J., Wennekes, F., Salomonsson, F., Hammar, M. & Streubel, K. Characterisation of n-InP/n-GaAs Wafer Fused heterojunctions. *Phys. Scr. T* **79**, 206–208 (1999).

[37] Wan, X. et al. Efficient and stable planar all-inorganic perovskite solar cells based on high-quality CsPbBr_3_ films with controllable morphology. *J. Energy Chem.* **46**, 8–15 (2020).

[38] Asahi, S., Kaizu, T. & Kita, T. Adiabatic two-step photoexcitation effects in intermediate-band solar cells with quantum dot-in-well structure. *Sci. Rep.* **9**, 1–8 (2019). 31133644 10.1038/s41598-019-44335-8 PMC6536537

[39] Panish, M. B. & Casey, H. C. Temperature dependence of the energy GaP in GaAs and GaP. *J. Appl. Phys.* **40**, 163–167 (1969).

[40] Geng, P. et al. A novel theoretical model for the temperature dependence of band gap energy in semiconductors. *J. Phys. D Appl. Phys.* **50** (2017).

[41] Asahi, S. & Kita, T. Strong voltage-boost effect in two-step photon-up conversion solar cells. In *2019 IEEE 46th Photovoltaic Specialists Conference (PVSC)* 2597–2599. 10.1109/PVSC40753.2019.8981300 (2019).

[42] Ali, A. et al. The electronic impact of Light-Induced degradation in CsPbBr_3_ Perovskite nanocrystals at Gold interfaces. *J. Phys. Chem. Lett.* **15**, 3721–3727 (2024). 38546374 10.1021/acs.jpclett.4c00139 PMC11017319

[43] Yuan, X. et al. Temperature-dependent photoluminescence of inorganic perovskite nanocrystal films. *RSC Adv.* **6**, 78311–78316 (2016).

[44] Seth, S., Mondal, N., Patra, S. & Samanta, A. Fluorescence blinking and photoactivation of all-inorganic perovskite nanocrystals CsPbBr_3_ and CsPbBr_2_I. *J. Phys. Chem. Lett.* **7**, 266–271 (2016). 26727624 10.1021/acs.jpclett.5b02639

[45] Pan, F. et al. Free and self-trapped exciton emission in perovskite CsPbBr_3_ microcrystals. *RSC Adv.* **12**, 1035–1042 (2022). 35425136 10.1039/d1ra08629d PMC8978929

[46] Cheng, H. et al. Understanding and minimizing non-radiative recombination losses in perovskite light-emitting diodes. *J. Mater.Chem. C* **10**, 13590–13610. 10.1039/d2tc01869a (2022).

[47] Meng, L. et al. Pure formamidinium-based Perovskite Light-Emitting diodes with high efficiency and low driving voltage. *Adv. Mater.* **29**, (2017). 10.1002/adma.201603826 27879016

[48] Sherkar, T. S. et al. Recombination in Perovskite Solar cells: significance of Grain boundaries, Interface traps, and defect ions. *ACS Energy Lett.* **2**, 1214–1222 (2017). 28540366 10.1021/acsenergylett.7b00236 PMC5438194

[49] Baruch, P., De Vos, A., Landsberg, P. T. & Parrott, J. E. On some thermodynamic aspects of photovoltaic solar energy conversion. *Sol. Energy Mater. Sol. Cells* **36**, 201–222 (1995).

[50] Löper, P. et al. Analysis of the temperature dependence of the open-circuit voltage. *Energy Procedia* **27**, 135–142 (2012).

[51] Wu, X. et al. ZnO electron transporting layer engineering realized over 20% efficiency and over 1.28 V open-circuit voltage in all-inorganic perovskite solar cells. *EcoMat* **4**, 1–11 (2022).

[52] Hombe, A., Saiki, S., Mori, T., Saito, Y. & Tanimoto, T. AlGaN as an electron transport layer for wide-bandgap perovskite solar cells. *Jpn. J. Appl. Phys.* **62** (2023).

[53] Dong, C., Han, X., Li, W., Qiu, Q. & Wang, J. Anti-solvent assisted multi-step deposition for efficient and stable carbon-based CsPbI_2_Br all-inorganic perovskite solar cell. *Nano Energy* **59**, 553–559 (2019).

