# Supplementary material for: Intraband transitions at a CsPbBr3/GaAs heterointerface in a two-step photon upconversion solar cell
Source: Sci Rep. 2024 Nov 6;14:26897. doi: 10.1038/s41598-024-78257-x (PMC11541914; doi:10.1038/s41598-024-78257-x)
Supplement: Supplementary file 1 — Supplementary Material 1 [file 41598_2024_78257_MOESM1_ESM.docx]

**Supplementary Information**

Intraband Transitions at a CsPbBr_3_/GaAs Heterointerface in a Two-Step Photon Upconversion Solar Cell

*Hambalee Mahamu^*^, Shigeo Asahi, and Takashi Kita.*

*Department of Electrical and Electronic Engineering, Graduate School of Engineering, Kobe University, 1-1 Rokkodai, Nada, Kobe 657-8501, Japan*

*^*^*Hambalee Mahamu: 203t268t@cloud.kobe-u.ac.jp

**S1. Simulation of Band Diagram using COMSOL Multiphysics**

In Device Structure of the TPU-SC, we described the TPU mechanisms using simulated band diagrams. COMSOL Multiphysics is powerful versatile software that can be used for simulation and modelling in engineering, physics, and mathematics. There are many calculation modules available on COMSOL Multiphysics. In our article, we used Semiconductor Module to calculate the band diagram of CsPbBr_3_/GaAs-based TPU-SCs. The simulation procedures are shown in the following:

First, we created a 1-dimensional model consisting of 3 parts. Material parameters were incorporated into each part. The 1-dimensional model, with labels, is shown in Fig. S1. The software provides some semiconductor libraries including GaAs data. Therefore, we incorporate GaAs data from the library into our model. Other semiconductor data is not available on the software. The data was acquired manually by referring to several publications. The necessary semiconductor parameters are shown in Table S1 with references.

Secondly, we added physics to our model. The physics we added includes generation rate in GaAs, direct and trap-assisted recombination in GaAs, ohmic contacts at GaAs and ZnO, and doping concentration of 1×10^19^ cm^–3^ (acceptor) and 1×10^18^ cm^–3^ (donor) for GaAs and ZnO respectively. The acceptor doping concentration of GaAs is the actual value of our GaAs wafer but the donor doping concentration was assumed because we had no information on the actual value. Here, COMSOL Multiphysics is embedded with algorithms that use equations related to solid-state physics to calculate specified solutions including band diagrams and quasi-Fermi levels of electrons and holes.

Thirdly, we set the calculation methods to MUMPS (Multifrontal Massively Parallel sparse direct Solver) together with nonlinear damping Newton’s method with highly nonlinear damping factor functions. We also set the meshes to ‘Extremely Fine’ and set 10^–9^ of tolerant error aiming to obtain reliable results.

For Fig. 1(b) in the article, the simulation was set to thermal equilibrium conditions without external applied voltage and illumination. For Fig. 1(c), the simulation was set to under constant illumination of 784-nm photons (100 mW/cm^2^) under thermal equilibrium conditions without external applied voltage. The 784-nm photoexcitation was expressed using extinction coefficients of GaAs as an input to the generation rate equation. It is noteworthy that the simulation of intraband excitation is not simply achieved in COMSOL Multiphysics due to several factors including the density of state of interface states, the electron density at the interface, the absorptivity, as well as the plasmonic effect that may happen as a result of electron accumulation. Therefore, we assumed additional electron concentrations (~10^15^ m^–3^s^–1^) in the conduction band (CB) of CsPbBr_3_. The electrons can be concluded as a result of adiabatic intraband excitation at the HI-I that excites photogenerated electrons in GaAs to the CB of CsPbBr_3_. Therefore, the quasi-Fermi level of electrons exhibits a slight increase depending on the electron concentration at the CB CsPbBr_3_. This procedure allows us to depict an increase in the electron quasi-Fermi level in Fig. 1(c).

In COMSOL Multiphysics, we did not incorporate tunneling effects. It is true that the possibility of the tunnelling effect cannot be ignored. For specific cases, if we consider the band diagram at an interface with a thin WGS layer, we would see an inclined shape of the CB of the WGS as a result of the built-in electric field which, in turn, results in a spike-like shape at the interface. The thickness of the spike-like shape depends on the electron affinity of the WGS and the NGS as well as the deepness of interface states from the CB minimum of the NGS.

Besides the possible tunnelling effect, we observed the thermal excitation phenomenon as described in Section S2 of Supplementary Information. Fig. S2 shows the temperature-dependence of the photocurrent which is evidence that the tunnelling process is dominated by the thermal excitation process. Since the tunnelling process does not depend on temperature, we would observe a constant photocurrent if the tunnelling process dominates the thermal activation. As a result, we conclude that even if the tunnelling process at the heterointerface cannot be neglected, it is dominated by thermal activation. Therefore, we did not take tunneling effects into account in COMSOL Multiphysics.


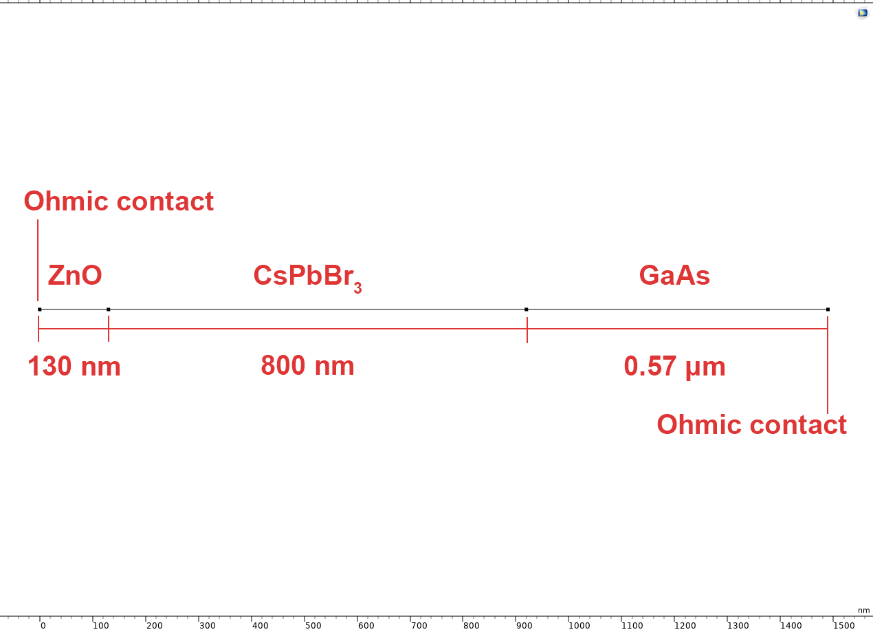


**Fig. S1** 1-dimensional model created in COMSOL Multiphysics software with labels indicating the applied metal contacts, thickness, and corresponding semiconductors.

| **Semiconductors** | **Parameters** | **Values** | **References** |
| --- | --- | --- | --- |
| CsPbBr_3_ | Bandgap (eV) | 2.3 | 1,2 |
|  | Electron affinity (eV) | 3.3 |  |
|  | Effective density of state for VB (cm^–3^) | 8.47×10^18^ | 3 |
|  | Effective density of state for CB (cm^–3^) | 4.94×10^17^ |  |
|  | Electron mobility (cm^2^/Vs) | 4500 |  |
|  | Hole mobility (cm^2^/Vs) | 4500 |  |
| ZnO | Bandgap (eV) | 3.37 | 4,5 |
|  | Electron affinity (eV) | 4.3 |  |
|  | Effective density of state for VB (m^–3^) | 1.13×10^25^ |  |
|  | Effective density of state for CB (m^–3^) | 2.94×10^24^ |  |
|  | Electron mobility (m^2^/Vs) | 60 |  |
|  | Hole mobility (m^2^/Vs) | 30 |  |

**Table S1** Some necessary semiconductor parameters used as inputs in COMSOL Multiphysics software with references

**S2. Temperature-dependent Short-circuit Current under 784-nm Photoexcitation**

We measured the *J*_SC_ as a function of the temperature for single-color excitation with *P*_Inter_ = 428 mW/cm^2^ (a photon flux of 1.69×10^22^ m^–2^s^–1^). The beam spot diameter on the SC was 1.2 mm. The SC was installed in the (evacuated) cryostat and we changed the temperature in the range from 280 to 300 K. The analysis was done using the Arrhenius equation, because we consider that the accumulated electrons overcome the heterointerface by thermionic emission.

The dashed line in Fig. S2 represents the result of fitting the data to the Arrhenius equation,

$$\text{J}_{\text{SC}}\text{ = A}\exp\left( \text{–}\frac{\text{E}_{\text{A}}}{\text{k}_{\text{B}}}\text{∙}\frac{\text{1}}{\text{T}} \right) \text{,} \text{ }$$

where *A* is a fitting parameter, *k*_B_ is the Boltzmann constant, *T* is the absolute temperature, and *E*_A_ is the thermal activation energy. Regarding *E*_A_, the observed IR-induced photocurrent enhancement (Figs. 6a and 6b in the article) would suggest an *E*_A_ that corresponds to the CB discontinuity at HI-I. However, the fit resulted in an *E*_A_ of 0.108 eV, which is significantly smaller than the estimated CB discontinuity of 0.770 eV. We ascribe the obtained value of *E*_A_ to an average energy level of occupied interface states at HI-II:

As indicated in the band diagram in Fig. 1b of the article, the CB discontinuity at HI-II has a barrier height of ~1.00 eV ($\text{χ}_{\text{ZnO}}$ is much larger than $\text{χ}_{\text{CsPbB}\text{r}_{\text{3}}}$). In this case, the electrons in the CB of CsPbBr_3_ should not need thermal energy to reach ZnO. Therefore, we consider the following process: First, the photogenerated electrons in the CB of GaAs accumulate at the interface states of HI-I. Then, the electrons are thermally excited to the CB of CsPbBr_3_ and transported to the front electrode. A fraction of these electrons relaxes from the CB to the interface states of HI-II and are trapped. These electrons need to be thermally activated to reach the CB of ZnO in order to be collected at the front electrode. This means that these electrons are first thermally excited at HI-I, and then are again thermally excited at HI-II. As a result, the physical meaning of the obtained value of *E*_A_ may be the energy difference between the CB minimum of ZnO and the average level of occupied interface states at HI-II.


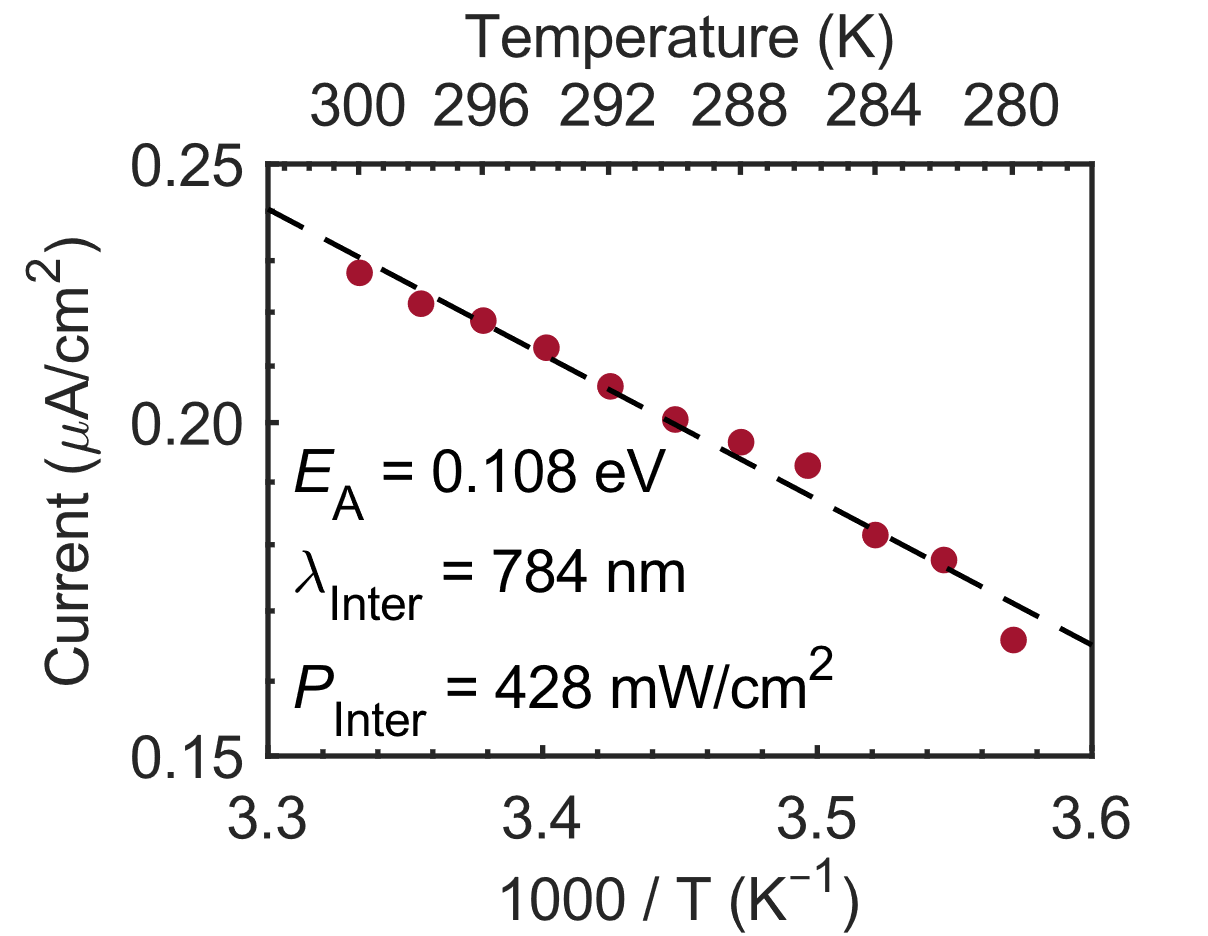


**Fig. S2.** Temperature dependence of the *J*_SC_ for single-color excitation with *P*_Inter_ = 428 mW/cm^2^. *E*_A_ and *λ*_Inter_ are the estimated thermal activation energy and the excitation wavelength, respectively.

**S3. Total Amount of 500-nm Photons Absorbed in Each Layer**

In EQE Spectra in the main article, we considered the fraction of 500-nm light absorbed in each semiconductor in our TPU-SC. In order to estimate the total amount of absorbed light in each layer, we employed the Beer–Lambert law,

*T =* exp(*–αx*),

where *T* is the transmittance, *α* is the absorption coefficient of the considered semiconductor, and *x* is the thickness of the layer. To simplify the calculation, we neglected the influences of reflection and scattering. The SEM image in Fig. S10 provided the thicknesses of the ZnO and CsPbBr_3_ layers (approximately 130 nm and 800 nm, respectively). Regarding the absorption coefficients, we used the values reported in the literature^6–8^. The result indicated in Fig. S3 reveals that 17% of the incident 500-nm photons are absorbed in ZnO and 82% are absorbed in CsPbBr_3_. The remaining 1% reaches GaAs. Therefore, the absorption of 500-nm photons in GaAs is negligible. Since more than 80% of the 500-nm photons are absorbed in CsPbBr_3_, the photon absorption by CsPbBr_3_ results in a significant reduction of the electron accumulation at HI-I as the energy of the photons for interband excitation increases.


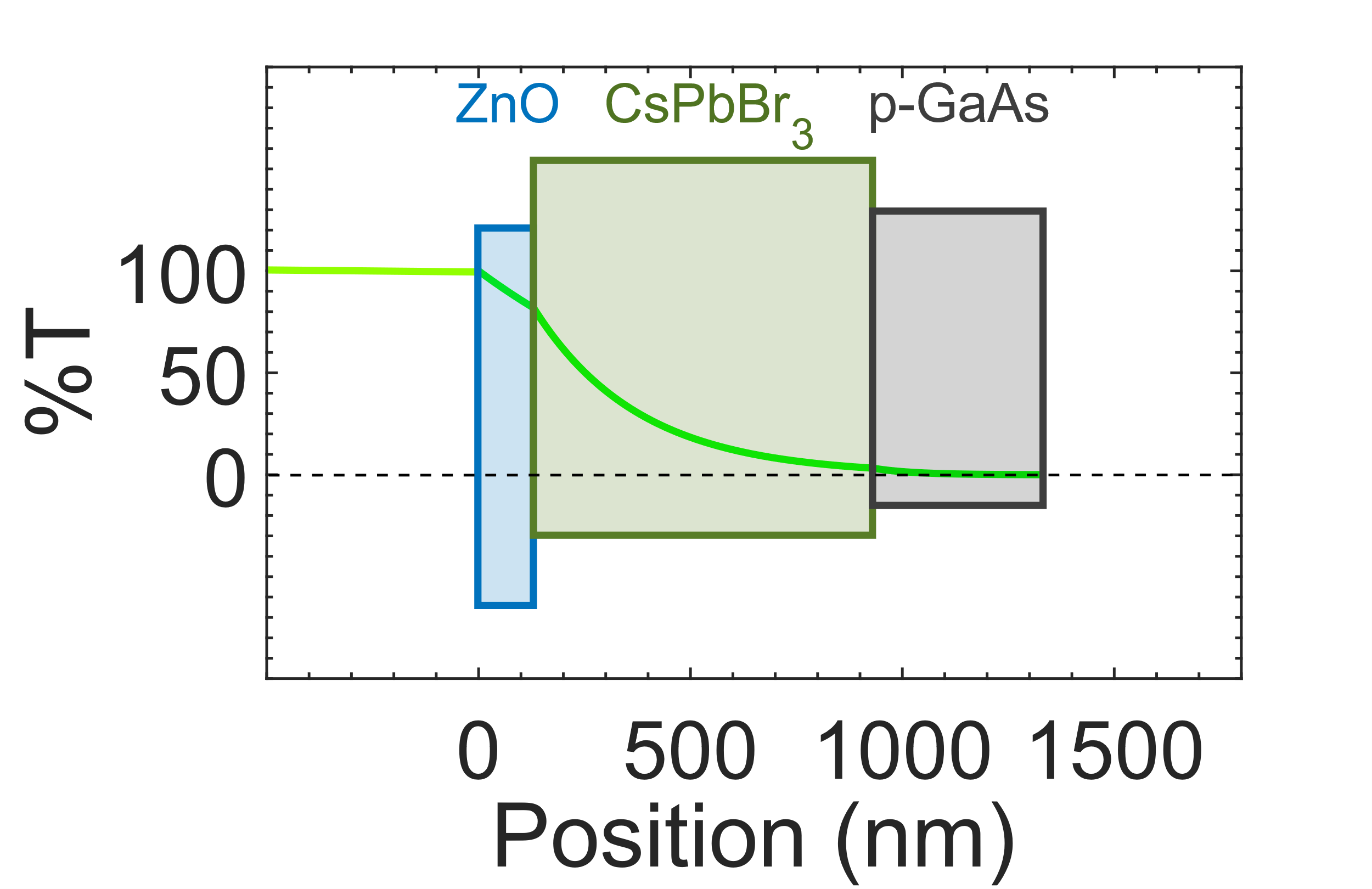


**Fig. S3** Transmission of 500-nm photons as a function of the position in the $\text{CsPbB}\text{r}_{\text{3}}$/GaAs-based two-step photon upconversion solar cell.

**S4. *JV* Characteristics**

The *JV* characteristics under different illumination conditions are shown in Fig. S4; the black, blue and dark red points represent the *JV* characteristics in the dark, under single-color excitation conditions (*P*_Inter_ = 101 mW/cm^2^), and under two-color excitation conditions (*P*_Inter_ = 101 mW/cm^2^ and *P*_Intra_ = 86 mW/cm^2^), respectively. Because the ZnO and CsPbBr_3_ layers are transparent for 784-nm photons, these photons are absorbed in the p-GaAs substrate and generate electrons and holes in the GaAs conduction band (CB) and valence band (VB), respectively. The photogenerated electrons drift toward HI-I, where they can accumulate at the interface states. The photogenerated holes drift to the rear electrode. While thermal excitation can in principle excite the electrons at HI-I to the CB of CsPbBr_3_, thermal activation hardly occurs since the energy difference between the CB minima of CsPbBr_3_ and GaAs is 0.77 eV, which is much higher than the thermal energy at room temperature (0.026 eV). This is the reason for the small magnitude of the detected photocurrent in the case of single-color excitation.

For two-color excitation, a slight photocurrent and photovoltage enhancement due to the additional IR photons can be confirmed. The IR-induced gain in the short-circuit current (Δ*J*_SC_) and the IR-induced gain in the open-circuit voltage (Δ*V*_OC_) are 0.011 µA/cm^2^ and 25 mV respectively.

The behavior of the open-circuit voltage (*V*_OC_) is interpreted as follows: The *V*_OC_ enhancement in the case of additional illumination with sub-bandgap photons (i.e., in the case of optically-induced intraband transitions) occurs because the 784-nm photons induce interband transitions only in the GaAs layer. When the electrons accumulated at HI-I are excited by the 1319-nm photons and reach the CB of CsPbBr_3_, the electron quasi-Fermi level shifts to a higher level and thereby increases the *V*_OC_. Thus, the observed positive Δ*V*_OC_ evidences an adiabatic intraband excitation process at HI-I.


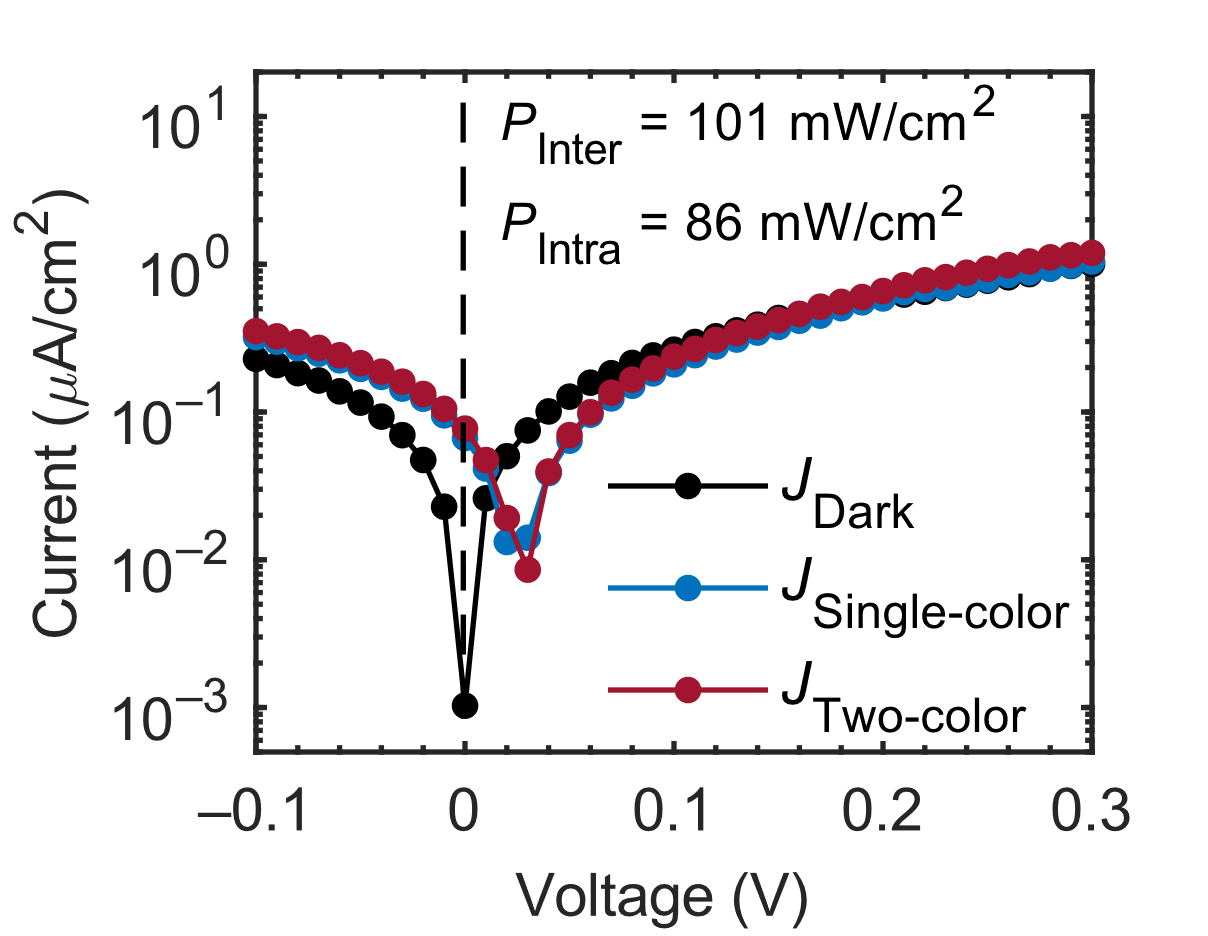


**Fig. S4.** Current–voltage (*JV*) characteristics of the CsPbBr_3_/GaAs-based TPU-SC. The *JV* curves were measured under different excitation conditions at room temperature. The black points indicate the *JV* characteristics in the dark. The blue and dark red points show the data for single-color excitation with *P*_Inter_ = 101 mW/cm^2^ and two-color excitation with *P*_Inter_ = 101 mW/cm^2^ and *P*_Intra_ = 86 mW/cm^2^, respectively.

**S5. Interband Excitation Power-dependent *V*_OC_**

The excitation power dependence of the *V*_OC_ for single-color excitation is provided in Fig. S5. The *V*_OC_ increases with *P*_Inter_, because the electron density in the CB of GaAs increases. Although the accumulated electrons are thermally excited to the CB of CsPbBr_3_ as shown in Fig. 5 of the article, the *V*_OC_ under single-color excitation conditions is determined by the quasi-Fermi-level splitting in GaAs, because in this case, the excitation at HI-I is a thermionic emission process.


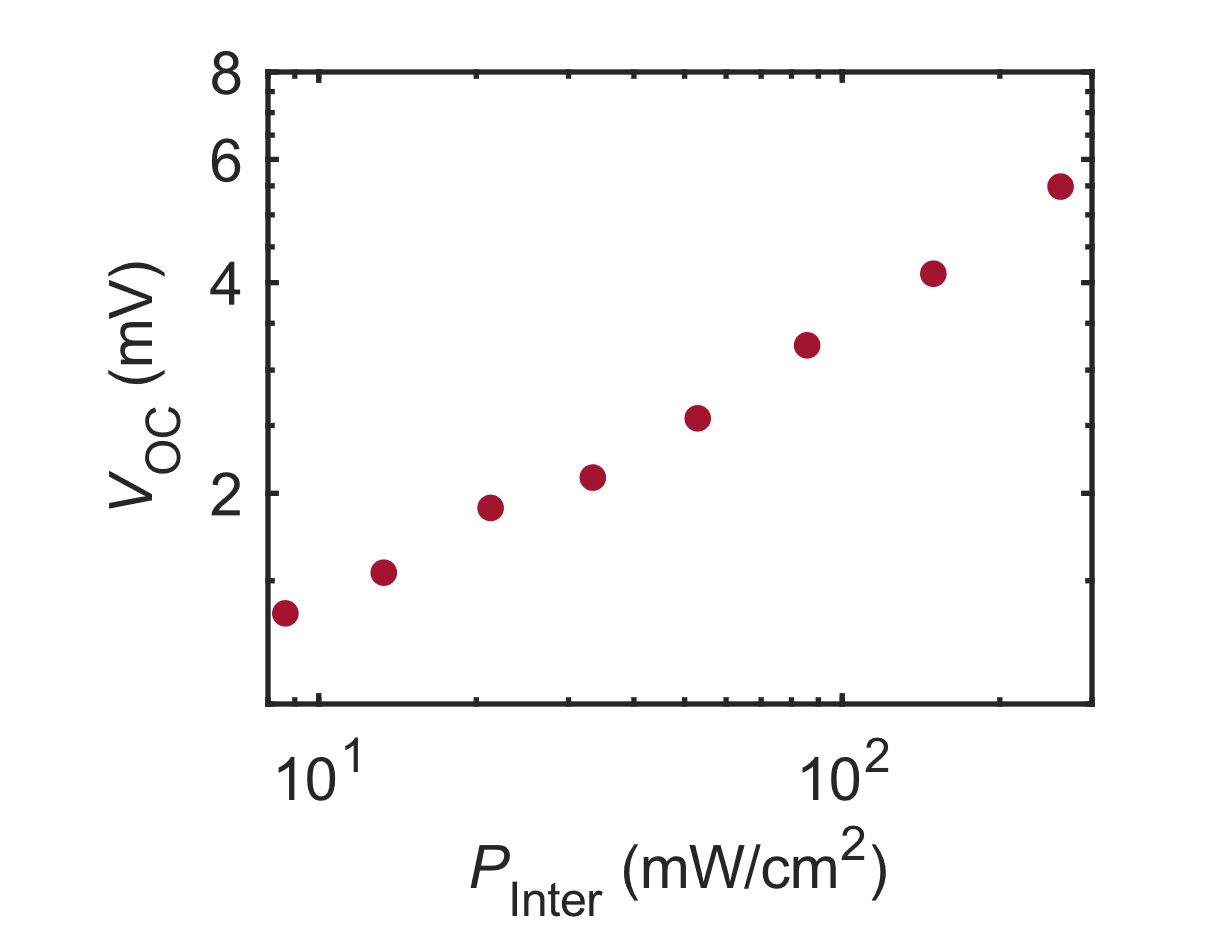


**Fig. S5.** The open-circuit voltage (*V*_OC_) for single-color excitation as a function of *P*_Inter_.

**S6. 1-sun Illumination *JV* Characteristic Curve**

Figure S6 shows the *JV* characteristic curve measured under the AM1.5 solar simulator. Overall, we obtained *J*_SC_ and *V*_OC_ of 0.44 µA/cm^2^ and 0.29 V providing a fill factor of 0.289. The device performance is substantially lower than the theoretical efficiency shown in Fig. 2, mainly because the observed photon upconversion is significantly smaller compared to the theoretical value. We expect the photon upconversion to be enhanced by employing quantum dots, as the electron confinements by quantum dots relax the optical selection rules for the intraband transition. Additionally, the quality of CsPbBr3 and the electron ZnO layer also need to be improved.


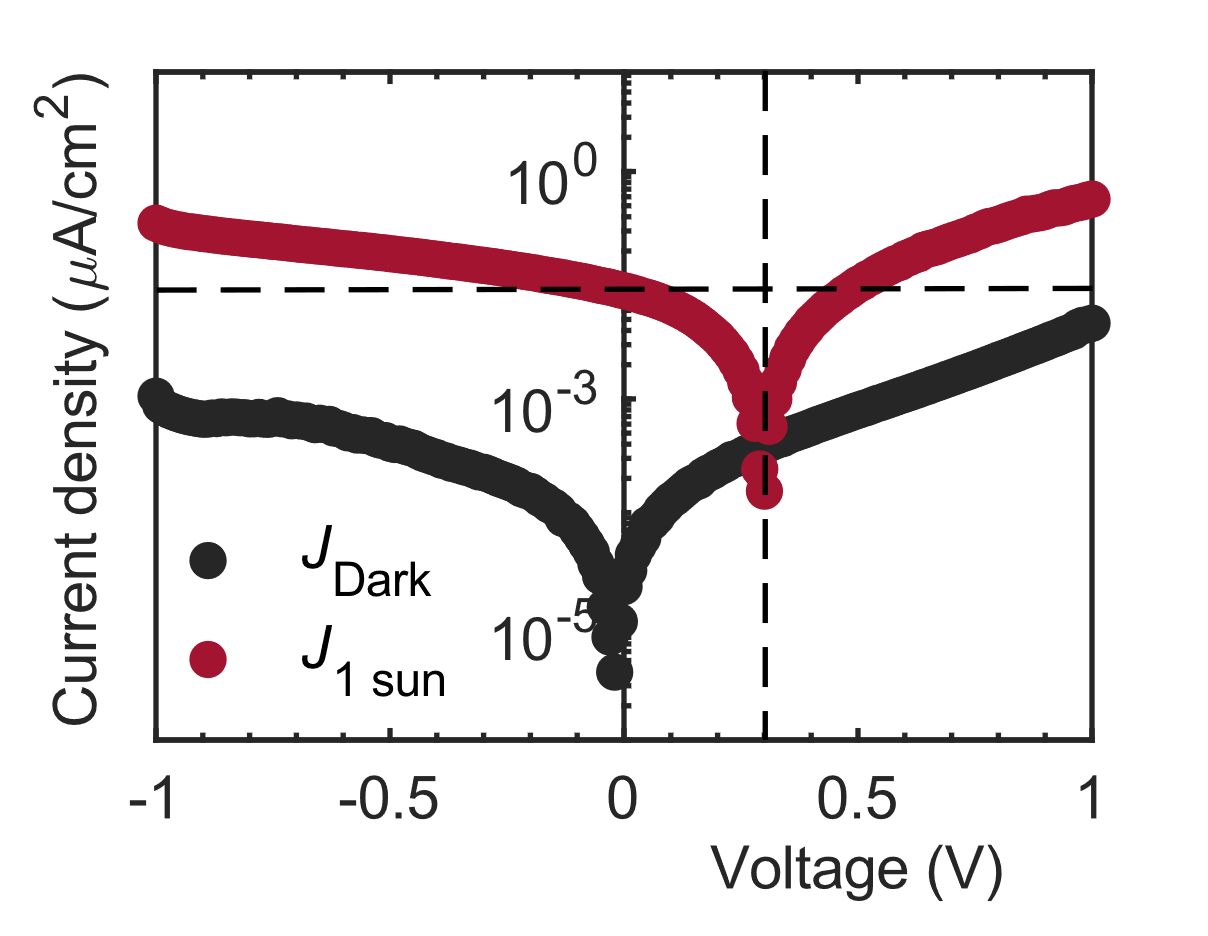


**Fig. S6.** The current-voltage characteristics measured under 1-sun illumination.

**S7. Absorption Spectrum of Four-month Left CsPbBr_3_**

Figure S7 shows the absorption spectrum of CsPbBr_3_ deposited on a glass substrate using the same recipe for CsPbBr_3_/GaAs TPU-SC fabrication. The CsPbBr_3_/glass sample was left in a humidity-controlled box for four months before it was carried out for measurement of the absorption spectrum. The spectrum shows a clear absorption edge with a strong exciton absorption peak at ~505 nm.


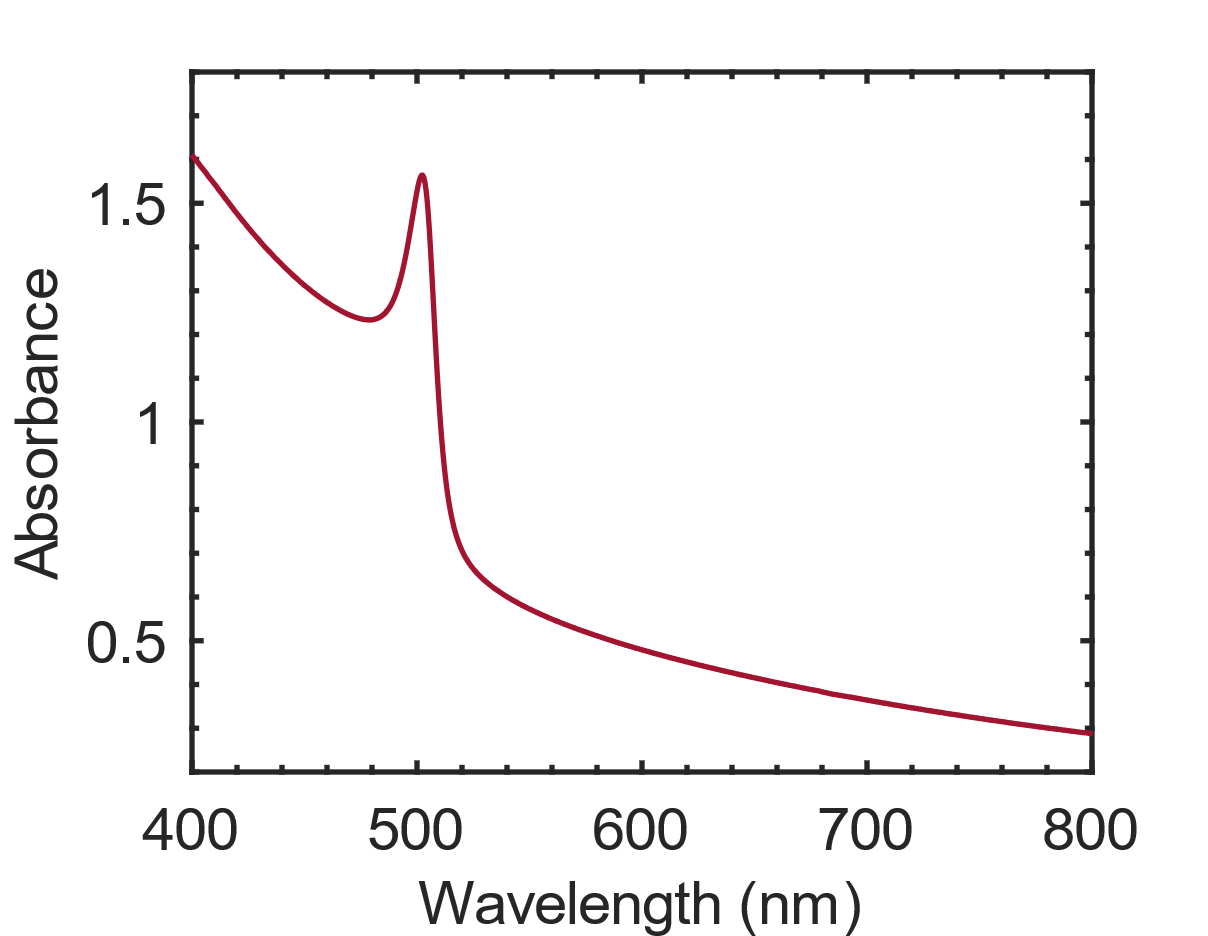


**Fig. S7** Absorption spectrum of a CsPbBr_3_/glass sample


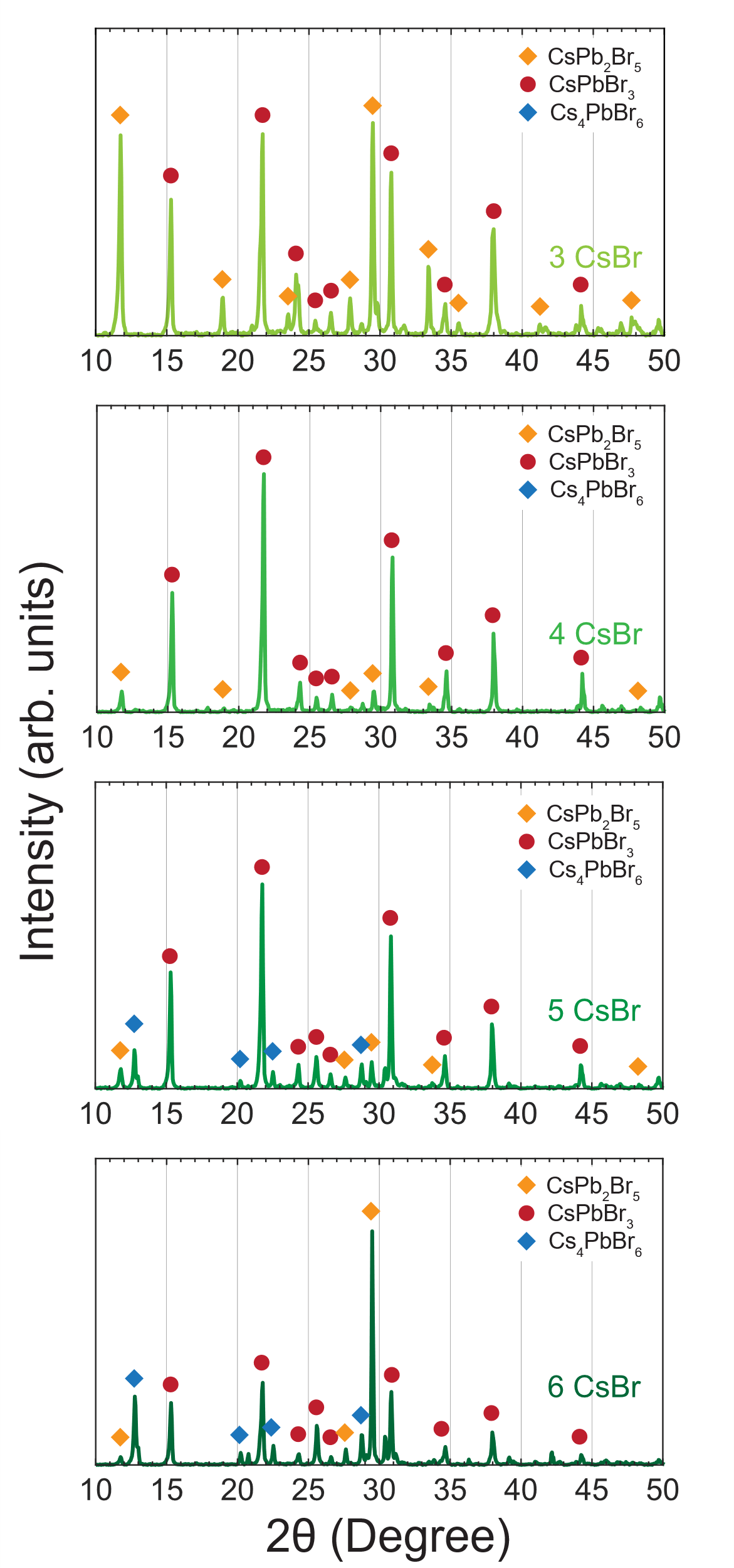
**S8. X-ray Diffractograms**

The measurement of the X-ray diffractograms was performed using a Rigaku Ultima IV automated multipurpose X-ray diffractometer. The X-ray photons with a wavelength of 1.541 Å (Cu K-*α*) were generated using an accelerating voltage of 40 kV. In the film prepared using three CsBr deposition cycles, the CsPb_2_Br_5_ phase (indicated by the orange squares) is dominant. The diffraction peaks corresponding to CsPbBr_3_ (indicated by dark red points) first increase with the number of deposition cycles. However, when more than four deposition cycles are used, the Cs_4_PbBr_6_ phase appears (indicated by the blue squares). Therefore, four deposition cycles are considered to be the optimum condition in our case.

**Fig. S8** X-ray diffractograms of CsPbBr_3_ films prepared on glass substrates using 3, 4, 5 and 6 CsBr deposition cycles. The diffraction intensity is shown on a linear scale. The orange and blue squares indicate the undesired derivative phases $\text{CsP}\text{b}_{\text{2}}\text{B}\text{r}_{\text{5}}$ and $\text{C}\text{s}_{\text{4}}\text{PbB}\text{r}_{\text{6}}$, respectively. The dark red points indicate the diffraction intensity of the CsPbBr_3_ perovskite phase.

**S9. Photoluminescence Spectrum**

**
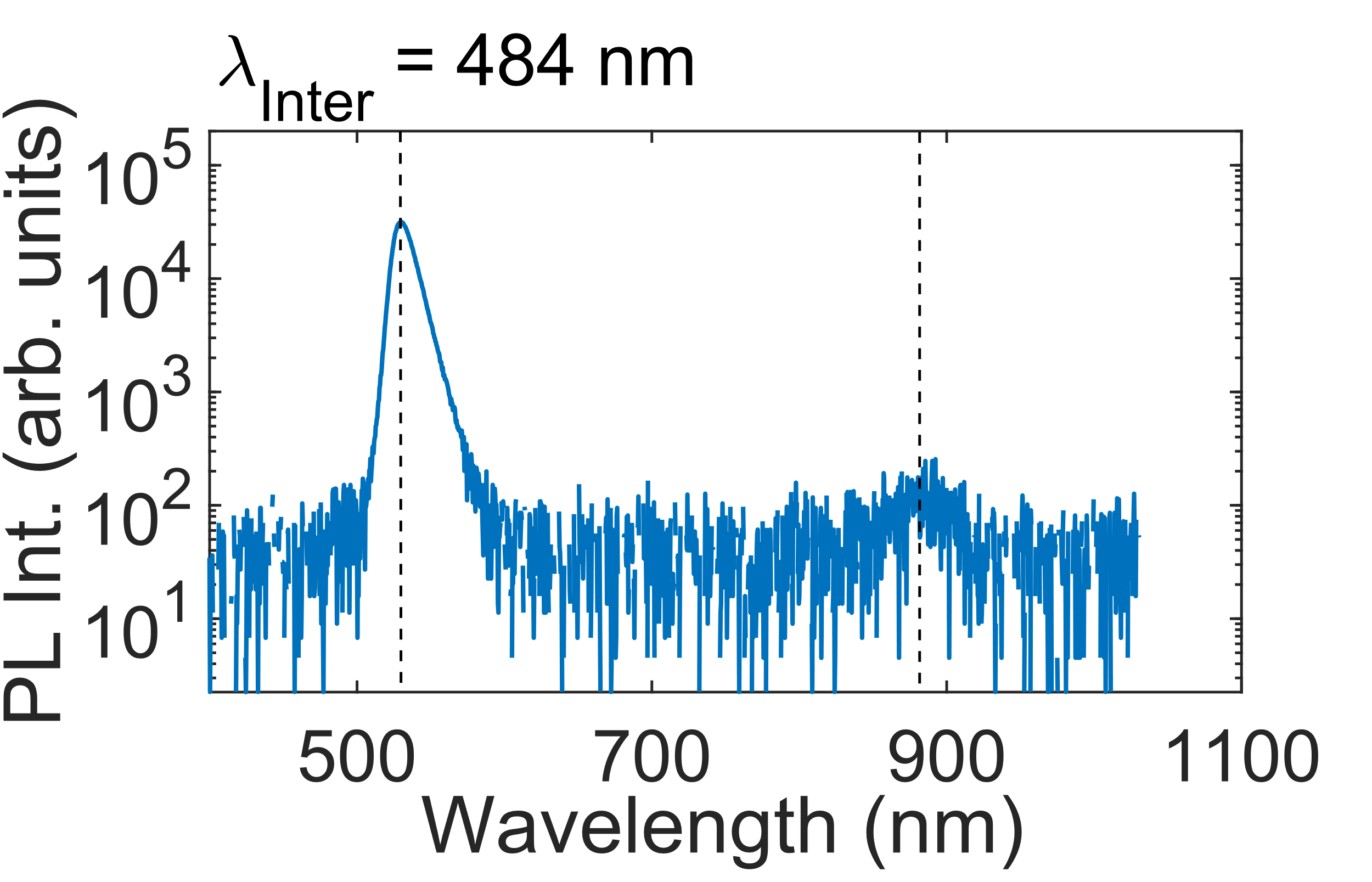
**

**Fig. S9** PL spectrum of CsPbBr_3_ on GaAs.

The room-temperature photoluminescence spectrum of CsPbBr_3_ on GaAs was measured by employing a continuous-wave solid-state laser operating at 484 nm. The excitation power was 39.5 mW. The 484-nm photons are mainly absorbed in the CsPbBr_3_ layer, but a small fraction also reaches the GaAs layer. Thus, the emission peak at about 535 nm, which corresponds to CsPbBr_3_, exhibits a much higher intensity than the emission peak of GaAs at about 870 nm. The spectrum was measured using an OceanOptics USB2000 spectrometer with a Sony ILX511B linear silicon CCD array (Ocean Insight).

**S10. Scanning Electron Microscopy Image**

**
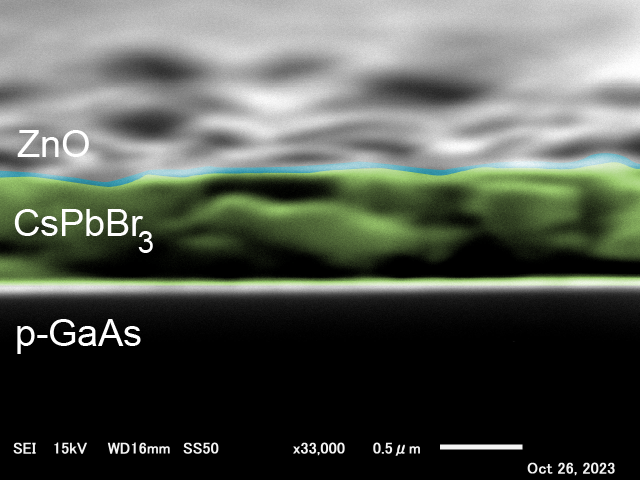
**

**Fig. S10** Cross-sectional scanning electron microscopy (SEM) image of the $\text{CsPbB}\text{r}_{\text{3}}$/GaAs-heterointerface.

For the characterization of the layer thicknesses, a ZnO/CsPbBr_3_/p-GaAs sample was installed in a scanning electron microscope (JSM-IT200 InTouchScope^TM^). Figure S3 shows a SEM image at a magnification of 33,000× for an accelerating voltage of 15 kV. The scale bar indicates that the thicknesses of ZnO and CsPbBr_3_ are approximately 130 nm and 800 nm, respectively. These two values were used to estimate the total amount of 500-nm photons absorbed in each layer.

**S11. Short-circuit Current and Open-circuit Voltage under Blue Light Excitation**

In addition to the *J*_SC_ and the *V*_OC_ measured as a function of 784-nm interband excitation power density, we also measured the *J*_SC_ and the *V*_OC_ as a function of 484-nm interband excitation power density. 18% of incident 484-nm photons are absorbed at ZnO layer while the remaining (83%) is absorbed by CsPbBr_3_. Therefore, for 484-nm photoexcitation case, there is significantly small electron accumulation at the HI-I. The accumulating electrons at the HI-I can be considered as a result of the electron diffusion process. Here, we observed increases in the *J*_SC_ and the *V*_OC_ with increasing 484-nm photoexcitation power density. This ensures that the CsPbBr_3_ works properly and can generate carriers under photoexcitations. The fitted value of power index (_n_) is larger than the one we observed in the case of 784-nm photoexcitation, which might be due to carrier extraction efficiency because the photogenerated carriers in ZnO and CsPbBr_3_ can reach the electrodes easier than the case of 784-nm photoexcitation.


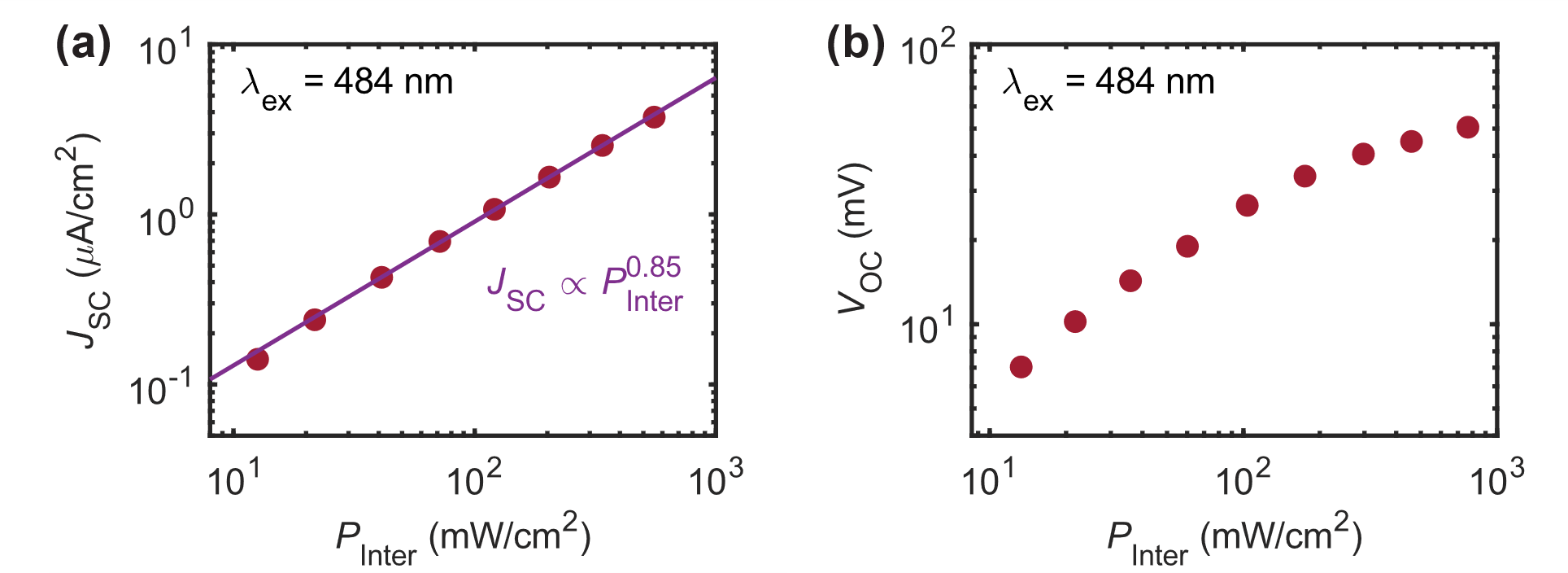


**Fig. S11** **(a)** *J*_SC_ as a function of 484-nm *P*_Inter_. **(b)** *V*_OC_ as a function of 484-nm *P*_Inter_.

**S12. Mechanism of *V*_OC_ reduction with temperature**

Carrier density is equivalent to the dark saturation current according to the detailed balance model following the equation below:

$$V_{\mathrm{OC}}=\frac{k_{B}T}{q} ln\left( \frac{F_{S}}{F_{C0}}+1 \right)$$

here, $F_{S}$ is incident photon numbers (equivalent to short-circuit current or the maximum photocurrent) and $F_{C0}$ are the intrinsic carriers at a constant temperature which is equivalent to the dark saturation current. Since the intrinsic carrier concentrations strongly depend on temperature, the dark saturation current exhibits strong temperature-dependent as well. The mobility, on the other hand, affects the position of the electron quasi-Fermi level. Ref. 9 provides information on the mobility of CsPbBr_3_ grown by epitaxial technique as a function of temperature. The mobility does not significantly change within the temperature ranges we used to measure the ∆*V*_OC_ in Fig. 9 of the main manuscript. This is similar to the bandgap which does not significantly change with temperature ^10^ unless the temperature is sufficiently high to transform CsPbBr_3_ crystals. Therefore, our opinion is that the changes in electron mobility do not significantly affect the ∆*V*_OC_. Hence, the conductivity should show good stability with temperature changes because the conductivity depends on mobility.

_______________________________________

**References**

1. Liao, G. *et al.* Enhanced charge extraction with all-carbon electrodes for inorganic CsPbBr_3_ perovskite solar cells. *Dalton Transactions* **47**, 15283–15287 (2018).

2. Bu, F. *et al.* Enhanced energy level alignment and hole extraction of carbon electrode for air-stable hole-transporting material-free CsPbBr_3_ perovskite solar cells. *Solar Energy Materials and Solar Cells* **205**, (2020).

3. Hossain, M. K. *et al.* Harnessing the potential of CsPbBr_3_-based perovskite solar cells using efficient charge transport materials and global optimization. *RSC Adv* **13**, 21044–21062 (2023).

4. Hossain, F. M. *et al.* Modeling and simulation of polycrystalline ZnO thin-film transistors. *J Appl Phys* **94**, 7768–7777 (2003).

5. Hirao, T. *et al.* Novel top‐gate zinc oxide thin‐film transistors (ZnO TFTs) for AMLCDs. *J Soc Inf Disp* **15**, 17–22 (2007).

6. Aspnes, D. E., Kelso, S. M., Logan, R. A. & Bhat, R. Optical properties of Al_x_Ga_1-x_ As. *J Appl Phys* **60**, 754–767 (1986).

7. Aguilar, O., de Castro, S., Godoy, M. P. F. & Rebello Sousa Dias, M. Optoelectronic characterization of Zn_1-x_Cd_x_O thin films as an alternative to photonic crystals in organic solar cells . *Opt Mater Express* **9**, 3638 (2019).

8. Ermolaev, G. *et al.* Giant and Tunable Excitonic Optical Anisotropy in Single-Crystal Halide Perovskites. *Nano Lett* **23**, 2570–2577 (2023).

9. Bruevich, V. *et al.* Intrinsic (Trap-Free) Transistors Based on Epitaxial Single-Crystal Perovskites. *Advanced Materials* **34**, (2022).

10. Mannino, G. *et al.* Temperature-Dependent Optical Band Gap in CsPbBr_3_, MAPbBr_3_, and FAPbBr_3_ Single Crystals. *Journal of Physical Chemistry Letters* **11**, 2490–2496 (2020).
